# Yishen Huashi Granules Ameliorated the Development of Diabetic Nephropathy by Reducing the Damage of Glomerular Filtration Barrier

**DOI:** 10.3389/fphar.2022.872940

**Published:** 2022-07-22

**Authors:** Tingting Zhao, Minyi Li, Qian Xiang, Beifeng Lie, Deqi Chen, Weiming Wang, Xuling Li, Tiancheng Xu, Xi Zhang, Yuntong Li, Ruixue Dong, Xinwen Du, Yilin Wang, Junzheng Yang, Bao He, Quan Zhu, Tingting Duan, Zhenghai Li, Youhua Xu

**Affiliations:** ^1^ State Key Laboratory of Quality Research in Chinese Medicines, Faculty of Chinese Medicine, Macau University of Science and Technology, Macao, China; ^2^ Institute of Consun Co., for Chinese Medicine in Kidney Diseases, Guangdong Consun Pharmaceutical Group, Guangzhou, China; ^3^ State Key Laboratory of Quality Research in Chinese Medicines, School of Pharmacy, Macau University of Science and Technology, Macao, China; ^4^ Department of Endocrinology, Zhuhai Hospital of Integrated Traditional Chinese and Western Medicine, Zhuhai, China; ^5^ Macau University of Science and Technology Zhuhai MUST Science and Technology Research Institute, Zhuhai, China

**Keywords:** diabetic neuropathy, proteinuria, Yishen Huashi, Chinese medicine, glomerular filtration barrier

## Abstract

**Background:** Diabetic nephropathy (DN) is one of the most common complications of diabetes and the primary cause of end-stage renal disease. At present, renin–angiotensin–aldosterone system (RAAS) blockers have been applied as first-class drugs to restrain development of DN; however, its long-term effect is limited. Recent evidence has shown definite effects of Chinese medicine on DN. Yishen Huashi (YSHS) granule is a traditional Chinese Medicine prescription that has been used in the clinic to treat DN, but its mechanism is not understood.

**Methods:** In the present study, both *in vitro* and *in vivo* studies were carried out. The DN model was induced by STZ in Wistar rats, and GEnC and HPC cell lines were applied in the *in vitro* study. Quality of YSHS was evaluated by LC-MS/MS. A metabolomic study of urine was carried out by LC-MS; influence of YSHS on composition of DN was analyzed by network pharmacology. Mechanism of the YSHS on DN was analyzed by Q-PCR, Western Blot, and multi-immunological methods.

**Results:** We found YSHS administration significantly reduced levels of HbA1c and mALB. Histopathological analysis found that YSHS preserved integrity of glomerular filtration barrier by preserving viability of glomerular endothelial cells and podocytes, inhibiting glomerular fibrosis, reducing oxidative stress damage, and enhancing cross-talk among glomerular endothelial cells and podocytes. Network pharmacology, differential metabolite analysis, as well as intracellular pathway experimental study demonstrated that the PI3K/AKT/mTOR signaling pathway played a pivotal role in it.

**Conclusion:** Our present findings supplied new understanding toward the mechanism of YSHS on inhibiting DN.

## 1 Introduction

With the increase in aging population, diabetes has become a global public health problem (Anders et al., 2018). Diabetic nephropathy (DN) is a common complication of diabetes mellitus and a common cause of end-stage renal disease (Gnudi et al., 2016). The early stage of DN features glomerular hyperfiltration, hypertrophy, microalbuminuria, basement membrane thickening (Giralt-López et al., 2020), mesangial expansion, and glomerular epithelial cell (podocyte) loss (Dai et al., 2017). Insulin resistance provoked hyperglycemia leads to increased levels of intracellular mitochondrial stress (Yaribeygi et al., 2020), and microalbuminuria (Papadopoulou-Marketou et al., 2018) has been considered the earliest marker of DN. Therefore, effective intervention in the early stage of DN is of great significance to delay the occurrence and development of nephropathy. Although much effort has been paid, the profit of the current strategy (Bai et al., 2019) on delaying development of DN is limited.

Yishen Huashi (YSHS) granule is a traditional Chinese Medicine prescription that has been used in the clinic to treat renal disease. According to its prescription, YSHS mainly comprises *Ginseng* (*Ren-Shen* in Chinese), *Astragali Radix* (*Huang-Qi* in Chinese), *Atractylodes macrocephala* (*Bai-Zhu* in Chinese), and *Poria cocos* (*Fu-Ling* in Chinese). Previously, we found a series of active components from the abovementioned herbal medicines on ameliorating diabetes-induced dysfunction of vascular endothelial cells and podocytes and were shown with effects on inhibiting progression of DN (Xu et al., 2011a; Xu et al., 2011b; Xu et al., 2015; Zhai et al., 2019; Zhu et al., 2019); most recently, we further found that oral administration with extract of *Astragali Radix* can enhance integrity of the gut barrier as well as blood–brain barrier and was shown with positive effects on inhibiting a series of diabetic complications (Wang et al., 2021; Xuling et al., 2022). Recently, an expert consensus from China Association of Chinese Medicine (The Panel of Yishen Huashi in China Association of Chinese Medicine, 2020) confirmed its definite effects on ameliorating kidney diseases exhibiting with proteinuria, but the underlying mechanism is still not understood.

To investigate the mechanism of YSHS on inhibiting development of DN, we designed this study. Both *in vivo* and *in vitro* experiments were involved. Our present study will supply direct evidence of YSHS on reducing proteinuria in DN.

## 2 Materials and Methods

### 2.1 Materials

JC-1 iodide (sc-364116) and primary antibodies for AKT (sc-514032), p-AKT (sc-8312), and DCFH (sc-359840) were purchased from Santa Cruz (Dallas, TX); 2-NBDG (N13195) was obtained from Life Technologies (Carlsbad, CA). Detection kits for MDA (A003-1-2), SOD (A001-3-2), BUN (C013-2-1), Cr (C011-2-1), mALB (E038-1-1), TP (A045-1-1), α1-MG (E017-1-1), and β2-MG (E015-1-1) were provided by Jiancheng (Nanjing, China). Detection kits for AGEs (CSB-E09413r), LPS (CSB-E14247r), HbA1c (CSB-E08140r), insulin (CSB-E05070r), and C-peptide (CSB-E05067r) were provided by CUSABIO (Wuhan, China). Irbesartan (zc-51741) was derived from Zzstandard (Shanghai, China). Detection kits for apoptosis (G003-1-3) were supplied by Beyotime (Shanghai, China). All other reagents were from commercial sources.

### 2.2 Yishen Huashi (YSHS) Qualification

**FIGURE 1:**
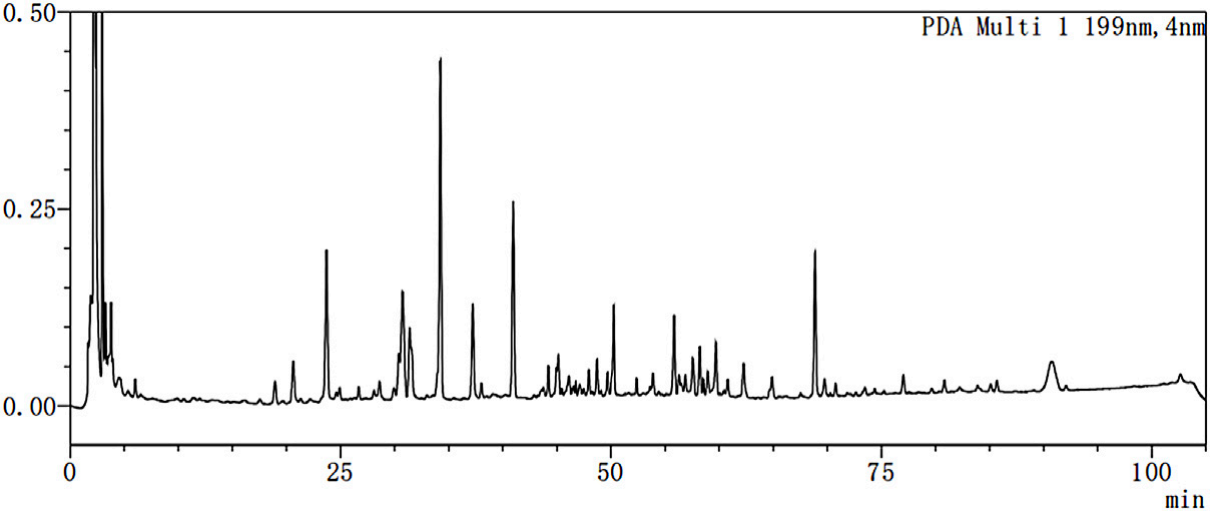
Fingerprint chromatogram of Yishen Huashi Extract.

YSHS (batch number: 20201101) was provided by Guangzhou Consun Pharmaceutical Co. Ltd (Guangzhou, China). Its quality was verified by HPLC detection (Figure 1). Calycosin isoflavone was quantified for controlling the quality of the granule. HPLC analyses of batch 20201101 sample showed a calycosin isoflavone content of 0.022 mg/mL. Please refer to the Supplementary Material S1 for details.

### 2.3 Animal Studies

All animal care and experimental design were approved by Macau University of Science and Technology. Male Wistar rats weighing 180–220 g were placed in specific pathogen-free (SPF) environment. The DN model was induced by intraperitoneal injection of streptozotocin (STZ) at the dose of 30 mg/kg once a week for 3 consecutive weeks. The model rats were fed with high-fat diet, while the control rats were fed with normal chow. Animals with blood glucose level >16.7 mmol/L were included to the subsequent experiments. DN rats were divided into five groups with six in each group: DN group, DN + YSHS (low dose, 1.3 g/kg/day), DN + YSHS (medium dose, 2.7 g/kg/day), DN + YSHS (high dose, 5.4 g/kg/day), and DN + Irbesartan (27 mg/kg/day) groups. Continuous gastric lavage administration of the drugs was given for 8 weeks, 6 days a week. After administration, urine is collected for 16 h. Animals were weighed prior to tissue collection. The animals were then anesthetized to collect blood.

### 2.4 Enzyme Immunoassay

Levels of AGEs (CSB-E09413r), LPS (CSB-E14247r), HbA1c (CSB-E08140r), MDA (A003-1-2), SOD (A001-3-2), Insulin (CSB-E05070r), and C-peptide (CSB-E05067r) were determined by kits according to the manufacturer’s protocols.

### 2.5 Western Blot

Protein extracted from kidney tissues was analyzed by WB. In general, proteins were electro-transferred to the PVDF membrane and incubated with primary antibodies overnight at 4°C. After washing with 1X TBST, the membranes were further incubated with secondary antibodies at room temperature for 1 h. Densitometric analysis of protein blots was analyzed using ImageJ software.

### 2.6 Transmission Electron Microscopy

The kidney morphology was observed by transmission electron microscopy (TEM). Freshly prepared kidney samples were immersed into 2.5% glutaraldehyde and fixed for at least 3 days. Sections at 70 nm were observed under a Hitachi TEM system.

### 2.7 Hematoxylin–Eosin Staining

After the treatment, the animals were sacrificed, and the kidneys were obtained after lavage with normal saline. Part of the kidney tissues was fixed in 10% neutral formaldehyde, embedded in paraffin, and pathological sections were prepared. Hematoxylin–eosin (H&E) staining was carried out according to the standard procedure.

### 2.8 Periodic Acid-Schiff Staining

After the treatment, the animals were killed, and the kidneys were obtained after lavage with normal saline. Pathological sections of the kidney were deparaffinized to water; after incubation with Schiff’s solution, sections were rinsed with running water; the nucleus was stained with Harris hematoxylin.

### 2.9 Immunohistochemistry

Kidney was fixed with 10% neutral formaldehyde. Sections were incubated with primary antibodies (1:100) overnight at 4°C, followed by secondary antibody incubation at 37°C for 30 min. Color was developed by DAB solution, and hematoxylin solution was applied for counterstaining.

### 2.10 Network Pharmacology

#### 2.10.1 Database and Analysis Software

Databases including TCMSP (http://lsp.nwu.edu.cn/tcmsp.php), Genecards (http://www.genecards.org), UniProt (https://www.uniprot.org), String (https://string-db.org), Bioconductor (http://www.bioconductor.org), Enrich (https://maayanlab.cloud/Enrichr/enrich), and DAVID (https://david.ncifcrf.gov/) were applied to collect data. Cytospase 3.8.0 software was used to analyze the data.

#### 2.10.2. Collection and Screening of Chemical Components

Chemical components in the drug were collected and screened through the TCM System Database (TCMSP) analysis platform, which is based on the absorption, distribution, metabolism, and excretion (ADME)–related indexes of the body to analyze the compounds and helps screen active compounds. According to Wu and Hu (2020) and Zhang et al. (2020), ingredients with oral bioavailability (OB) ≥ 30% and drug-like properties (DL) ≥ 0.18 were believed to have potential action and thereafter will undergo the screening procedure.

#### 2.10.3 Target Collection and Network Construction and Protein Interaction Analysis

The compound–active ingredient–target protein network was constructed by the UniProt database, and the protein–protein interaction (PPI) analysis was analyzed by the String database.

#### 2.10.4 GO Analysis

GO (Gene Ontology) analysis of molecular function, biological process, and cell composition for Yishen Huashi Granules was completed *via* ID correspondence or the sequence annotation method.

#### 2.10.5 KEGG Analysis

In order to study the synergistic effect of Yishen Huashi Granules in ameliorating diabetic nephropathy at the signal pathway level, KEGG analysis was conducted.

### 2.11 *In Vitro* Studies

Glomerular endothelial cells (GEnCs) were derived from ScienceCell, and human podocyte (HPC) cells were a gift from Professor Dingkun Gui (Department of Nephrology, Shanghai Jiao Tong University Affiliated Sixth People’s Hospital, Shanghai, China).

Cell viability was determined by the CCK-8 kit. In brief, cells were plated in 96-well plates. After drug incubation for different time periods 24 or 48 h at 37°C, the wells were added with CCK-8 solution. Two hours later, the optical density was measured at 450 nm using a Multimode microplate reader (Molecular Devices, U.S.).

### 2.12 Immunofluorescence

Cells were incubated with drugs for 24 h and then fixed with 4% paraformaldehyde solution. For permeabilization, cells were incubated with 0.1% Triton X-100 PBS solution for 10 min at room temperature. After washing with PBS, unspecific binding sites were blocked with blocking buffer (5% BSA in PBS) for 60 min at room temperature. Then, the cells were incubated with primary antibodies overnight at 4°C, followed by secondary antibody for 1 h in the dark at room temperature. Nuclei were counterstained by DAPI. Images were taken with a confocal laser scanning microscope (Leica TCS SP8, Germany) under standardized conditions and analyzed with ImageJ software.

### 2.13 Flow Cytometry

Cells were seeded in a 6-well plate dish at 3 × 10^5^ cells/mL. For the combination group, cells were pre-treated with drug for 24 h and then incubated with DCFH, JC-1, FITC/PI, or mitochondria deep red for 30 min in the dark. After harvesting, the cells were further washed with PBS three times and finally analyzed with a flow cytometer (BD FACS Aria III).

### 2.14 Metabolomic Study of the Urine

The cryopreserved urine sample was defrosted and shaken at 4°C. 50 µL of each sample was transferred into 2 mL centrifuge tubes. 150 µL 2-chlorophenylalanine was added with 50% acetonitrile as the internal standard, shaken for 5 min, and mixed well and centrifuged at 4 °C for 10 min at 12,000 rpm. The supernatant was filtered through a 0.22-µm membrane to obtain the prepared samples for LC-MS; the samples are used for LC-MS detection. Chromatographic separation of the urine sample was accomplished in an Thermo Ultimate 3,000 system equipped with an ACQUITY UPLC^®^ HSS T3 (150 × 2.1 mm, 1.8 µm, Waters) column maintained at 40°C. The temperature of the autosampler was 8°C. Gradient elution of analytes was carried out with 0.1% formic acid in water (C) and 0.1% formic acid in acetonitrile (D) or 5 mM ammonium formate in water (A) and acetonitrile (B) at a flow rate of 0.25 mL/min. Injection of 2 μL of each sample was carried out after equilibration. An increasing linear gradient of solvent B (v/v) was used as follows: 0–1 min, 2% B/D; 1–9 min, 2%–50% B/D; 9–12 min, 50%–98% B/D; 12–13.5 min, 98% B/D; 13.5–14 min, 98%–2% B/D; 14–20 min, 2% D-positive model (14–17 min, 2% B-negative model). The ESI-MSn experiments were executed on the Thermo Q Exactive Focus mass spectrometer with the spray voltage of 3.5 kV and −2.5 kV in positive and negative modes, respectively. The analyzer scanned over a mass range of m/z 81-1 000 for full scan at a mass resolution of 70 000. Data-dependent acquisition (DDA) MS/MS experiments were performed with HCD scan. The normalized collision energy was 30 eV.

### 2.15 Statistical Analysis

The results were presented as mean ± SEM. Data were analyzed by one-way analysis of variance (ANOVA) followed by GraphPad prism 7.00. *p* < 0.05 was considered statistically significant.

## 3 Results

### 3.1 Yishen Huashi Inhibited Progression of Diabetic Nephropathy

**FIGURE 2:**
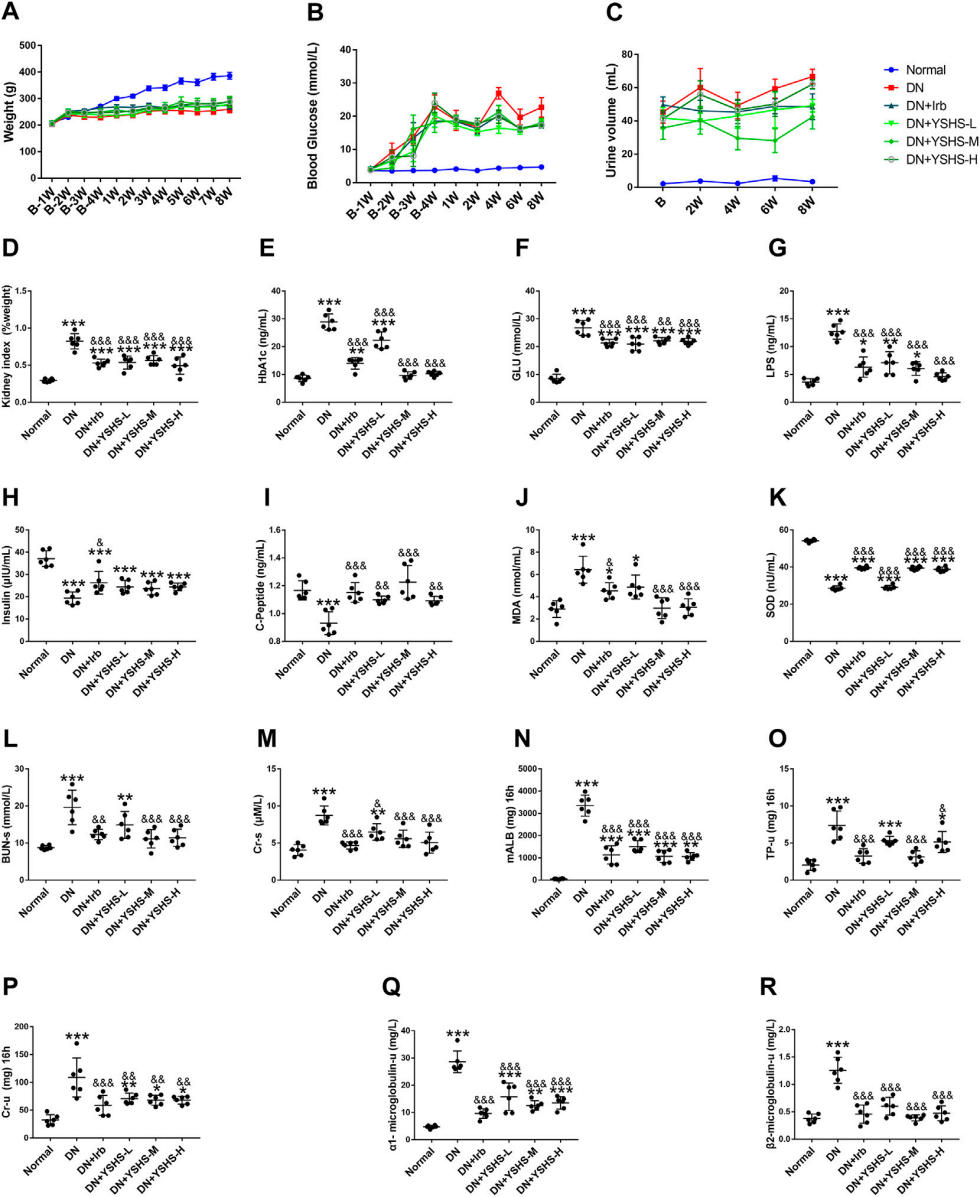
Effect of YSHS on biochemical parameters in DN rats. Changes in body weight **(A)**, blood glucose **(B)**, and urine volume **(C)** throughout the experimental period. Changes in kidney index **(D)** and parameters of blood HbA1c **(E)**, glucose **(F)**, LPS **(G)**, insulin **(H)**, C-peptide **(I)**, MDA **(J)**, and SOD **(K)** at the end of the experiment. Changes of serum BUN-s **(L)**, Cr-s **(M)**, and mALB (N), TP-u **(O)**, Cr-u **(P)**, α1-MG **(Q),** and β2-MG **(R)** within urine at the end of the experiment. Values are presented as means ± SEM. *p < 0.05, **p < 0.01, and ***p < 0.001, vs. Normal; ^&^ *p* < 0.05, ^&^ *p* < 0.01, and ^&&&^ *p* < 0.001, vs. DN.

The DN rats were orally administrated with the drug for 8 weeks. As shown in Figures 2A–D, although YSHS has no significant effects on body weight, fasting blood glucose, and urine volume, it significantly reduced kidney index. The plasma LPS (Figure 2G) was decreased by YSHS. Although the level of insulin (Figure 2H) was not significantly increased by the YSHS, both HbA1c (Figure 2E) and C-peptide (Figure 2I) were ameliorated by the drug, suggesting YSHS possessed beneficial effects against insulin resistance in DN. Moreover, levels of plasma BUN and Cr as well as those of urine mALB, TP-u, Cr-u, α1-MG, and β2-MG were significantly reduced by YSHS in DN rats (Figures 2L–R).

### 3.2 Yishen Huashi Ameliorated Histopathological Changes of the Kidney in Diabetic Nephropathy Rats

**FIGURE 3:**
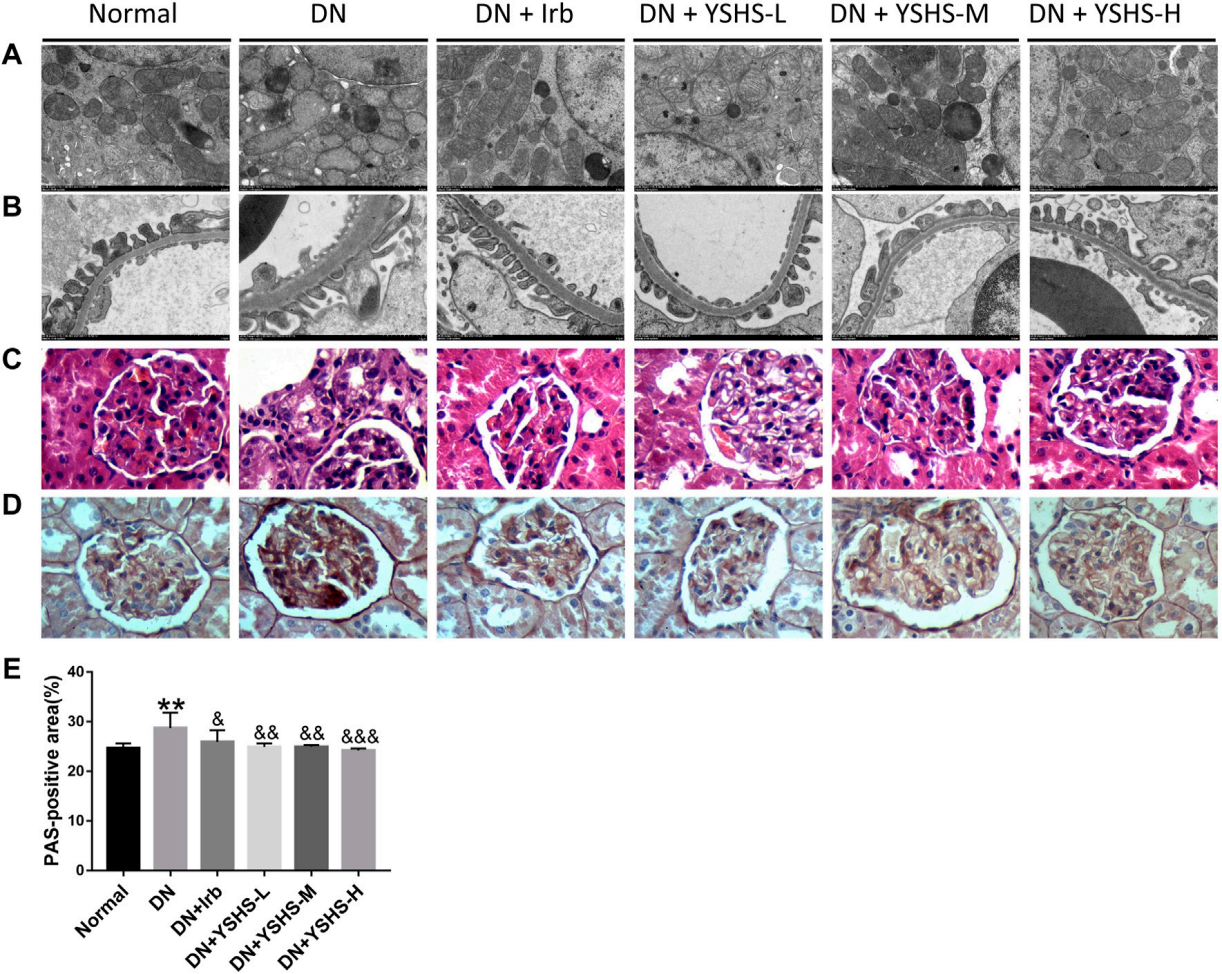
Effects of YSHS on histopathologic changes of DN rats. TEM **(A,B)**, H&E **(C)**, and PAS **(D)** staining of YSHS in different groups on DN. **(E)** PAS-positive area was analyzed by the IPP software. Values are presented as means ± SEM. **p < 0.01, vs. Normal; ^&^ *p* < 0.05, ^&&^ *p* < 0.01, ^&&&^ *p* < 0.001, vs. DN.

To verify the effects of YSHS on restoring DN damage, kidney tissues were observed by TEM, histopathological examination, and Periodic Acid-Schiff (PAS) staining. As shown in Figures 3A,B, the basement membrane of the filtration barrier in the model group was unevenly thickened; the number of mitochondria within glomerular endothelial cells was increased but were shown with edema and mitochondrial cristae decrement; in podocytes, the pseudopod was partially fused and some were not found. For H&E and PAS staining (Figures 3C–E), the glomerular basement membrane thickness and glomerular sclerosis were obviously observed in DN rats, while YSHS oral administration significantly preserved histological integrity of the nephron and inhibited glomerular sclerosis.

### 3.3 Yishen Huashi Modulated Expression of Fibrosis-Related Gene Expression in Diabetic Nephropathy Rats

**FIGURE 4:**
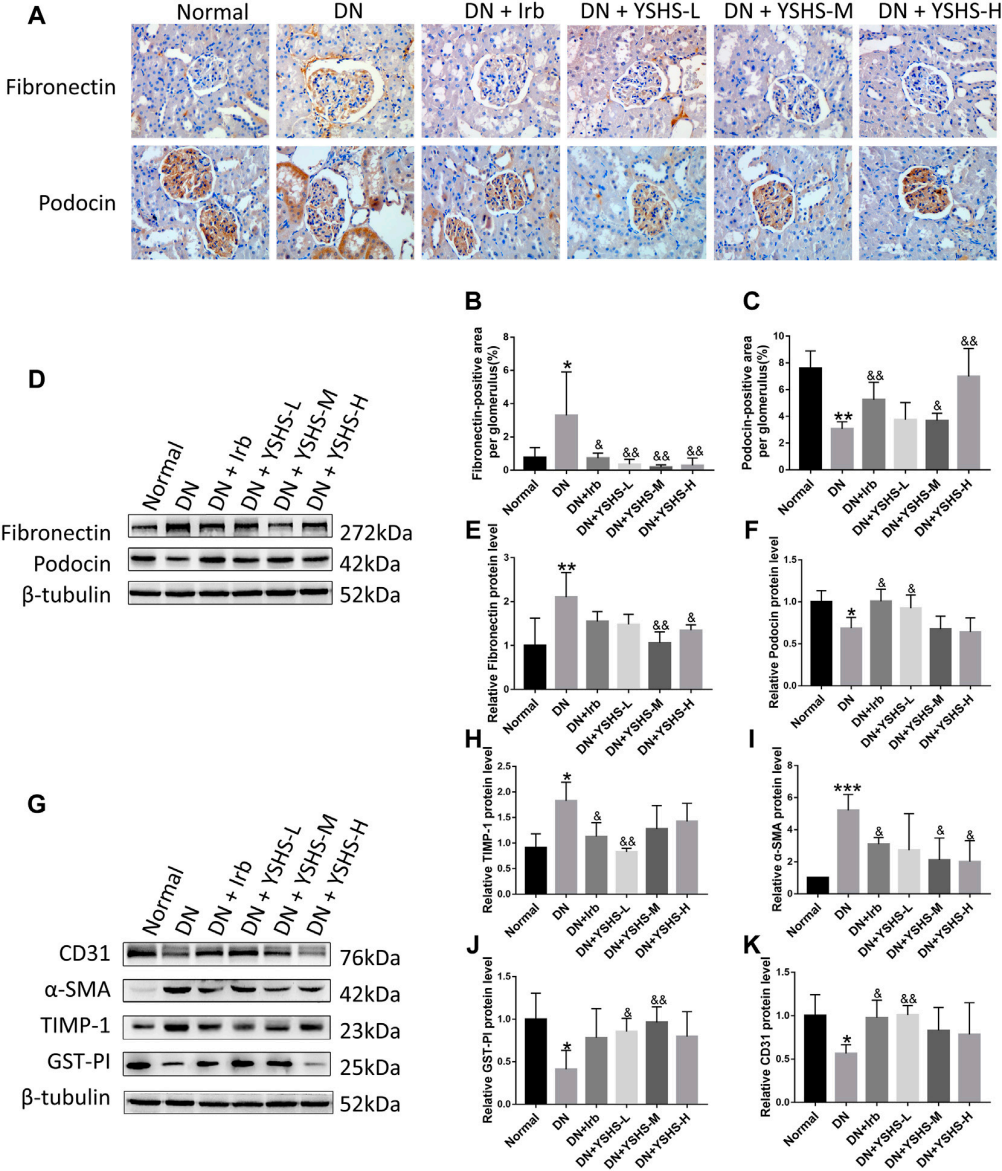
Effects of YSHS on renal fibrosis in DN rats. Podocin and fibronectin expression was detected by immunohistochemical staining **(A)**. IHC was quantitated for fibronectin **(B)** and podocin **(C)** using the IPP software. Podocin and fibronectin expression was determined by WB **(D)**. Relative expression of fibronectin **(E)** and podocin **(F)** was calculated using ImageJ software. Protein expression of CD31, *α*-SMA, TIMP-1, and GST-PI in the DN rat was determined by WB **(G)**. Relative expression of TIMP-1**(H)**, *α*-SMA **(I)**, GST-PI **(J)**, and CD31 **(K)** was analyzed by ImageJ software. Values are presented as means ± SEM. *p < 0.05, **p < 0.01, ***p < 0.001, vs. Normal; ^&^ *p* < 0.05, ^&&^ *p* < 0.01, vs. DN.

To explore the molecular mechanism of YSHS on restoring glomerular sclerosis, the expression of fibrosis-related genes was determined by both immunohistochemistry and Western blot. As shown in Figures 4A–C, positive particles of fibronectin protein were highly expressed in DN rat kidneys and podocin protein was lowly expressed in the glomerulus compared with normal; while both irbesartan and YSHS administration significantly reversed their expression. Similar results were found in the WB experiment (Figures 4D–F). *α*-SMA and TIMP-1 are two important markers that imply activity of the fibroblast; while GST-Pi and CD31 are two modulators that protect the cells against cytotoxic factors. In the present study, we found YSHS significantly reduced over-expressed *α*-SMA and TIMP-1 while increased GST-Pi and CD31 expressions (Figures 4G–K).

### 3.4 Network Pharmacology Analysis and Differential Metabolite Analysis of Urine

**FIGURE 5:**
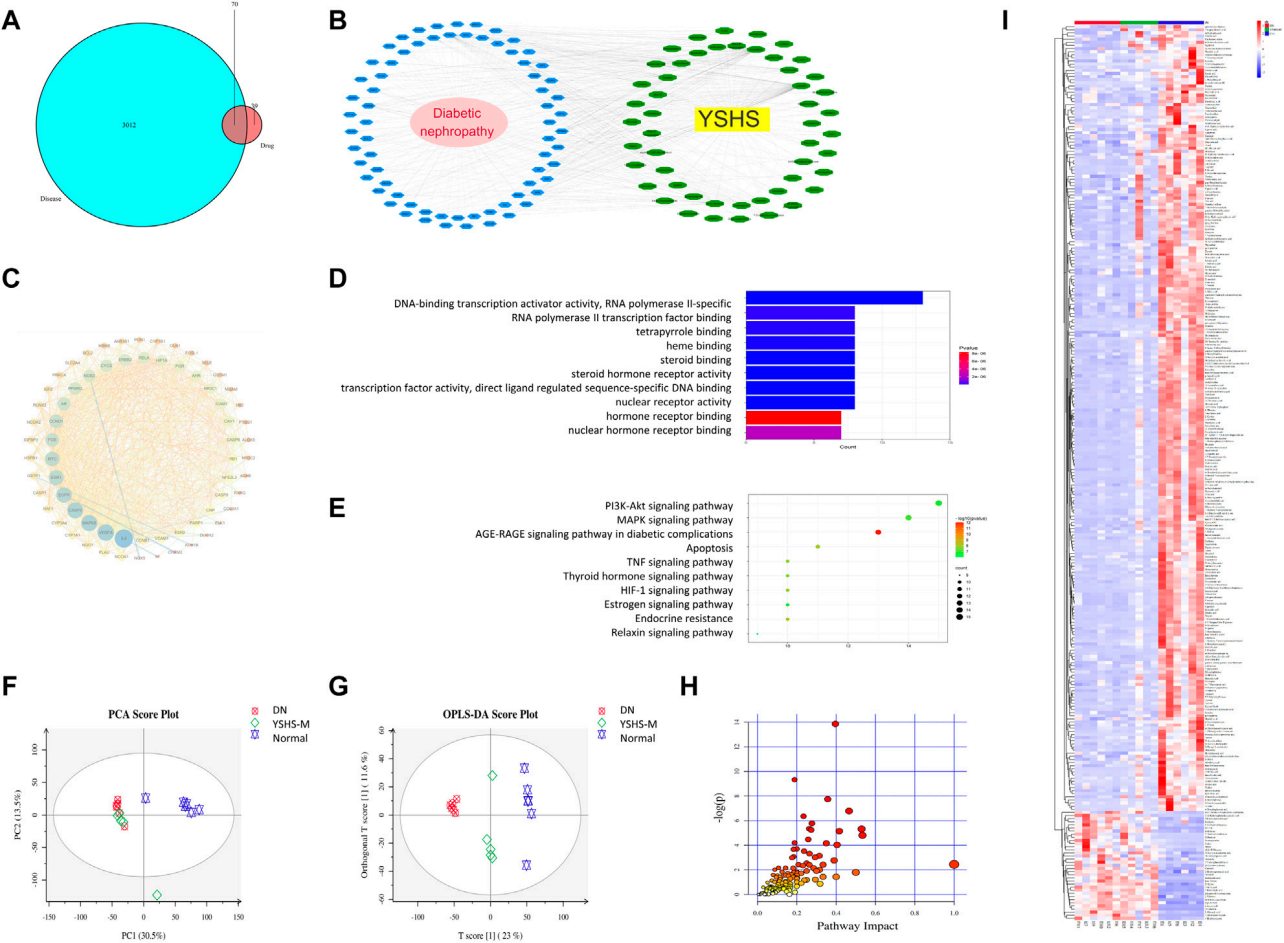
Network pharmacology analysis and differential metabolite analysis of urine. Venn diagram of diseases and YSHS drugs **(A)**. Compound target disease (CTD) network of YSHS **(B)**; the green circles represent compound targets, and the blue circles represent diseases. The PPI network **(C)** was sorted in descending order from the bottom of the figure in a circular layout, on the basis of the number of degrees; the size of the target and disease nodes reflects the number of degrees. Biological processes related to targets of YSHS **(D)**. Pathway analysis using the Kyoto Encyclopedia of Genes and Genomes (KEGG) **(E)**. Metabolomic analysis of rat urine: Separation of rat urine in a PCA score plot **(F)**; loading S-plot generated by OPLS-DA analysis of the metabolome in rat urine **(G)**; differential metabolite pathway analysis **(H)** differential metabolite heat map of the urine among groups **(I)**.

To explore the potential ingredients and molecular targets of YSHS that have an effect on DN, we conducted a network pharmacological analysis. We identified 82 potentially active compounds (Supplementary Table S1) and 109 gene targets corresponding to those potentially active compounds in YSHS. We also searched for 3,712 related DN target genes. A Venn intersection target diagram was built, and 70 common targets were identified (Figure 5A).

For a comprehensive identification of the mechanism of YSHS on DN, we constructed a network between YSHS-active components, the corresponding targets, and DN-related genes (Figure 5B). In combination with high-frequency node analysis in the PPI network (Figure 5C), we found IL6 (interleukin 6), VEGFA (vascular endothelial growth factor A), MAPK8 (mitogen-activated protein kinase 8), and CASP3 (caspase 3) are at the center of the network. GO (Figure 5D) and KEGG (Figure 5E) analyses were conducted for pathway enrichment to identify the relevant pathways and functions based on putative targets. Functional analysis revealed that the MAPK signaling pathway and the phosphatase binding and protein serine/threonine kinase (PI3K/AKT) signaling pathway significantly enriched the DN-related pathways. Moreover, functional analysis data revealed that these putative targets not only modulated cell proliferation, apoptosis, growth, and inflammatory response but also tuned the PI3K/AKT signaling pathways.

To further analyze metabolic changes of the drug on DN rats, metabolite analysis of the urine in rats receiving medium-dose YSHS was conducted. As shown in Figures 5A,F,G significance among the DN, YSHS, and normal groups was observed. The separation of YSHS-treated samples from corresponding controls and models was evident in the score scatter plot of the PCA on rat urine. There were 287 specific metabolic biomarkers that distinguished among the YSHS, DN, and normal groups (Figure 5H), and a total of 171 pathways were involved (Supplementary Table S2); the genes were binned into various metabolic pathways like phenylalanine metabolism, arginine and proline metabolism, glucagon signaling pathway, insulin secretion, citrate cycle (TCA cycle), insulin resistance, HIF-1 signaling pathway, insulin signaling pathway, etc. Overall, these results suggest association of the glucagon signaling pathway, insulin secretion, and insulin signaling pathway with DN. The heat map for these metabolites in the YSHS, DN, and control groups is shown in Figure 5I, and compared with the normal DN group, 964 metabolites were increased and 2,797 metabolites were decreased in the YSHS group, while 605 metabolites were increased and 34 metabolites were decreased in the YSHS group.

### 3.5 PI3K/AKT/m-TOR Signaling Pathway Participated in Yishen Huashi-Modulated Diabetic Nephropathy Rats

**FIGURE 6:**
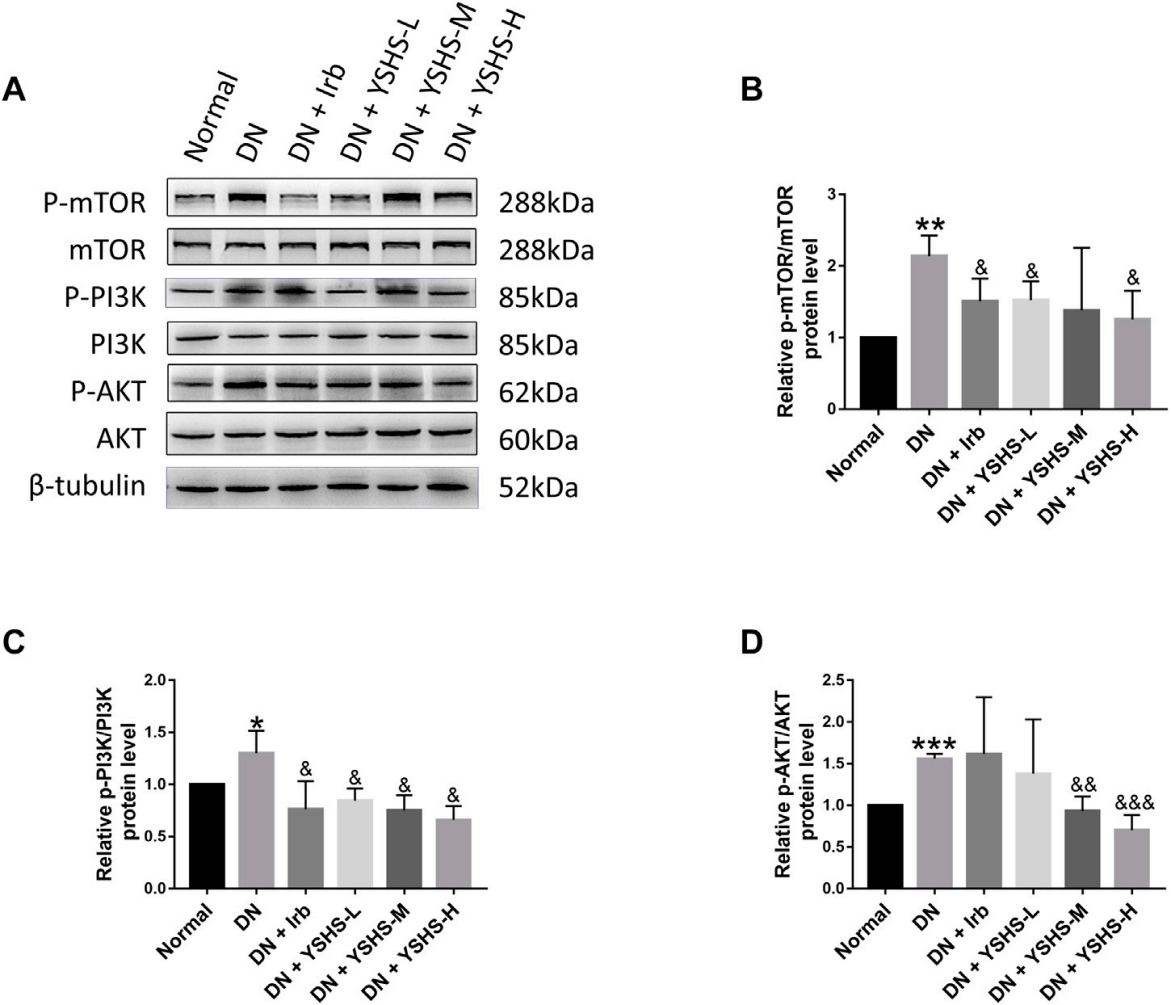
Western blot analysis of PI3K/AKT/m-TOR signaling pathways **(A)**. Relative expression and activation of mTOR **(B)**, PI3K **(C)**, and AKT **(D)** was analyzed by the ImageJ software. Values are presented as means ± SEM. *p < 0.05, **p < 0.01, ***p < 0.001, vs. Normal; &*p* < 0.05, &&*p* < 0.01, &&&*p* < 0.001, vs. DN.

To verify the abovementioned obtained signaling pathway in YSHS-modulated effects in the kidney of diabetic rats, expression and activation of some proteins were determined by WB. As shown in Figures 6A–D significant over-expression and activation of mTOR, AKT, and PI3K was observed in DN rats, and YSHS administration reduced their expression to the normal levels.

### 3.6 Yishen Huashi Increased Viability of Glomerular Endothelial Cells and Human Podocytes Cells Induced by High-Glucose and High-Insulin

**FIGURE 7:**
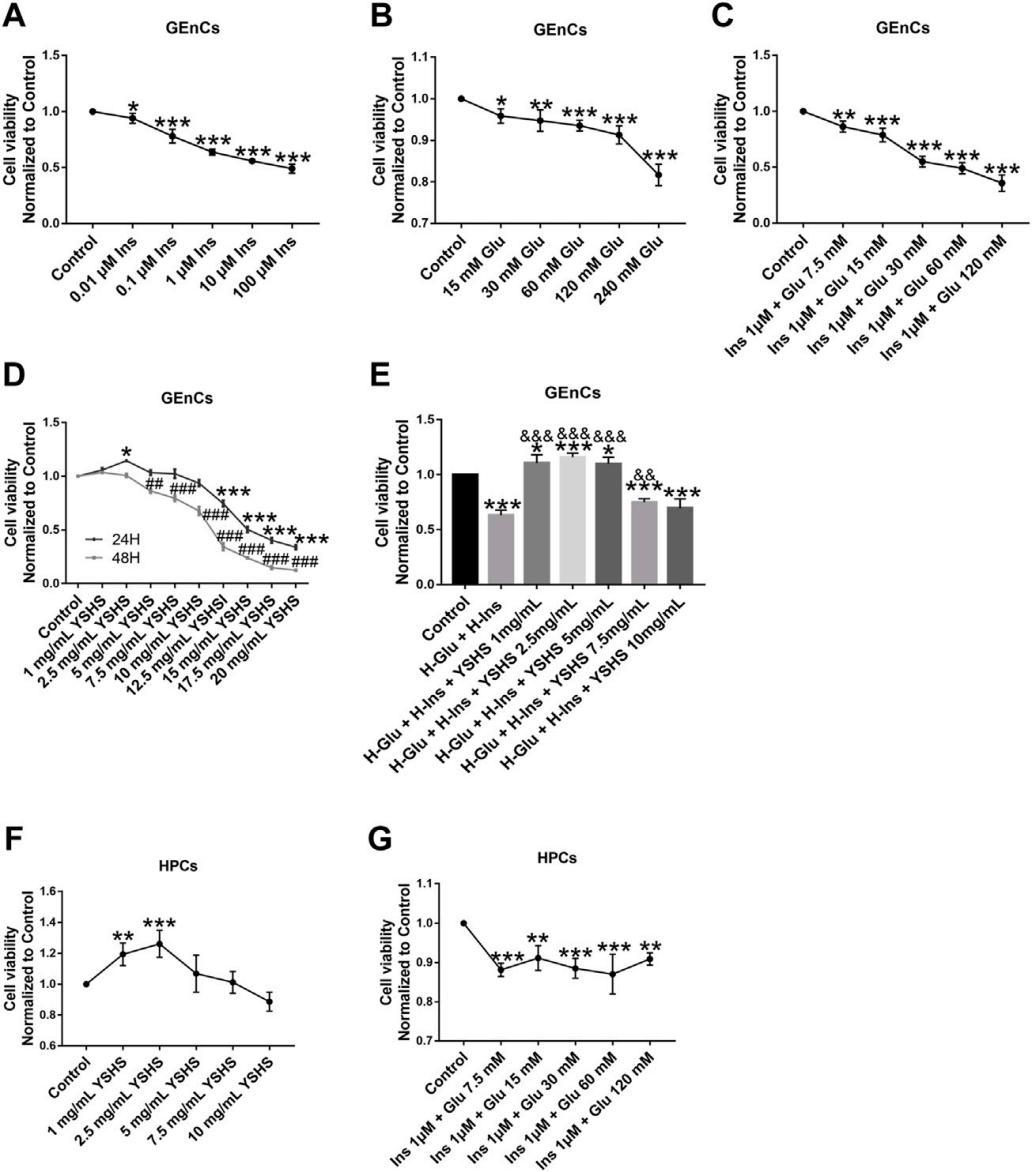
Influence of YSHS on viability of the GEnC and HPC cells. Viability analysis of GEnCs on drug administration **(A–E)**; viability analysis of HPCs on drug administration **(F,G)**. Data are expressed as the mean ± SEM. Each experiment was repeated at least three times. **(A–C,F,G)** *p < 0.05, **p < 0.01, ***p < 0.001, vs. control. **(D)**, *p < 0.05, **p < 0.01, ***p < 0.001, vs. 24 h control; ^##^ *p* < 0.01, ^###^ *p* < 0.001, vs. 48 h control. **(E)**, *p < 0.05, **p < 0.01, ***p < 0.001, vs. control. **(D)**, ^&^ *p* < 0.05, ^&&^ *p* < 0.01, ^&&&^ *p* < 0.001, vs. H-Glu + Ins.

To study the direct effect of YSHS on the cells, glomerular endothelial cells (GEnCs) and podocytes (HPCs) were applied in the following study. We first determined the influence of YSHS on viability of the cells. For this, we constructed the DN model by high-glucose (30 mM) + high insulin (1 μM) in GEnCs (Figures 7A–C). For viability assay, we found YSHS at 2.5 mg/mL showed protective effects on increasing viability of both GEnCs and HPCs (Figures 7D–G).

### 3.7 Oxidative Stress in Yishen Huashi-Modulated Cell Viability

**FIGURE 8:**
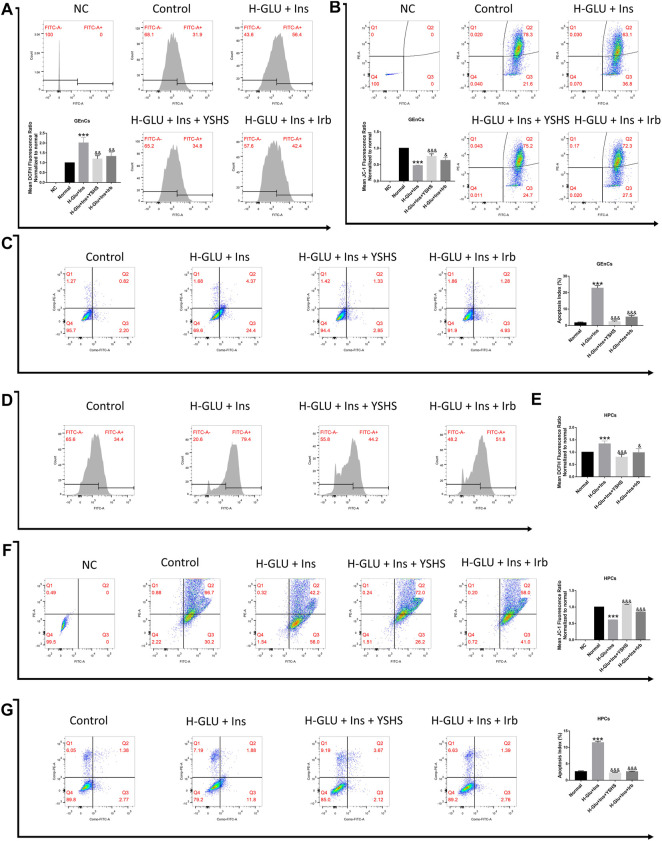
Effect of YSHS on oxidative stress, mitochondrial membrane potential (Δψm), and apoptosis in GEnC cells and HPC cells. Intracellular ROS levels within GEnC **(A)** and HPC **(D,E)** cells were measured by flow cytometry. JC-1 levels on GEnCs **(B)** and HPCs **(F)** cells were measured by flow cytometry. Apoptosis of GEnC **(C)** and HPC **(G)** cells were measured by flow cytometry. Values are presented as means ± SEM. *p < 0.05, **p < 0.01, ***p < 0.001, vs. control; ^&^ *p* < 0.05, ^&&^ *p* < 0.01, ^&&&^ *p* < 0.001, vs. H-Glu + Ins.

Oxidative stress and its related mitochondrial function play a pivotal role in cell viability. We found hyperglycemia and insulin resistance induces oxidative stress and production of reactive oxygen species (ROS), and blocking ROS production with YHSH administration could significantly ameliorate the effects of hyperglycemia and insulin resistance in both GEnCs (Figure 8A) and HPCs cells (Figures 8D,E). In the present study, mitochondrial membrane potential (ΔΨm) was also ameliorated by detection of JC-1 (Figure 8B) and DCFH (Figures 8C,F) in GEnC and HPC cells.

### 3.8 Yishen Huashi Preserved the Integrity of the Glomerular Filtration Barrier

**FIGURE 9:**
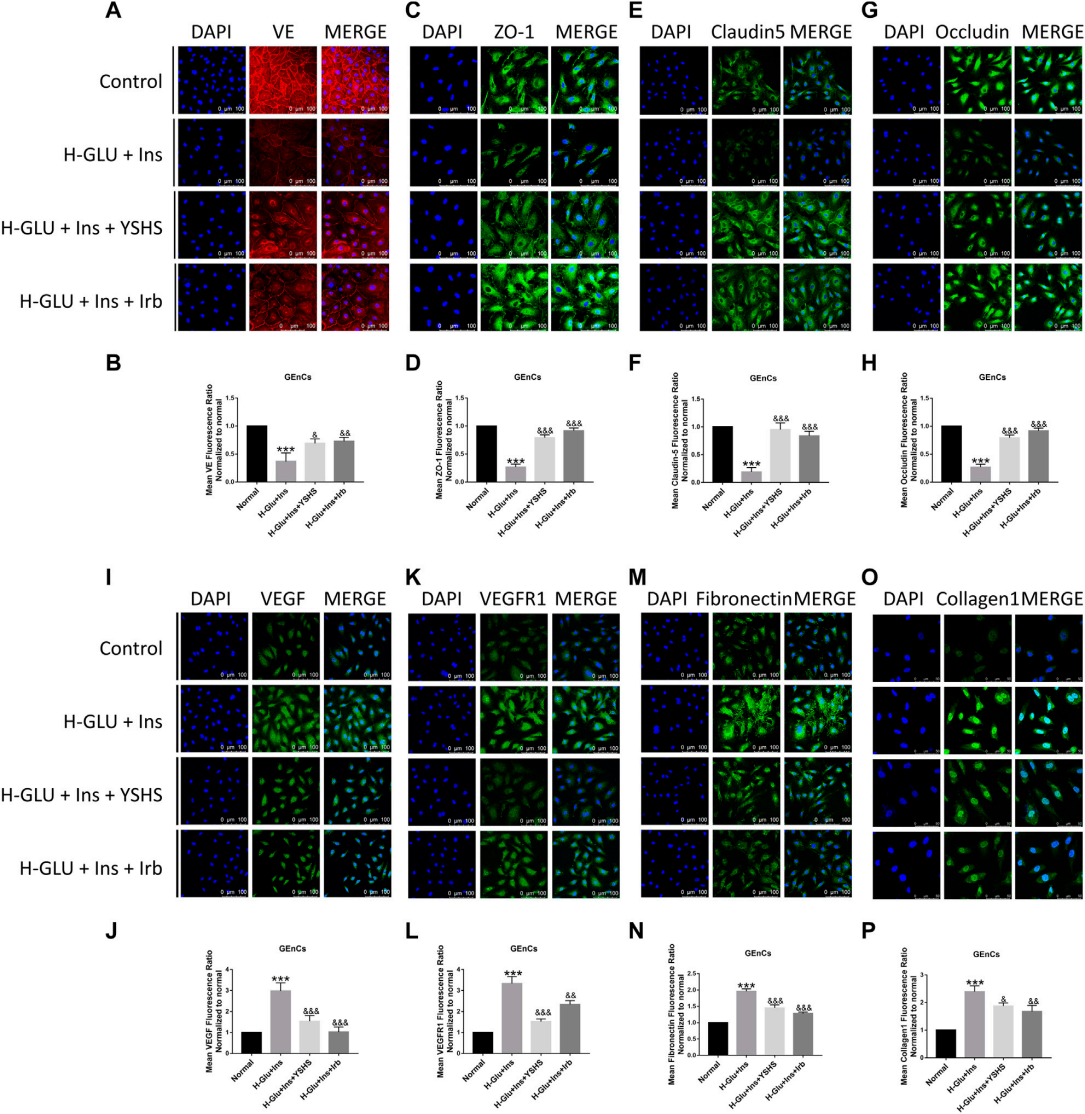
Effect of YSHS on the glomerular filtration barrier in GEnC cells. Immonofluorescence assay for GEnCs tight junction protein VE **(A)**, ZO-1 **(C)**, claudin-5 **(E)**, and occludin **(G)** under a laser scanning confocal microscope; and relative fluorescence intensity for them were determined **(B,D,F,G)**. YSHS decreased VEGF **(I)**, VEGFR1 **(K)**, fibronectin **(M)**, and collagen1 (O) expressions, and relative fluorescence intensity for them **(J)**, **(L,N)**, and **(P)** were determined. Values are presented as means ± SEM. ***p < 0.001, vs. control; ^&^ *p* < 0.05, ^&&^ *p* < 0.01, ^&&&^ *p* < 0.001, vs. H-Glu + Ins.

**FIGURE 10:**
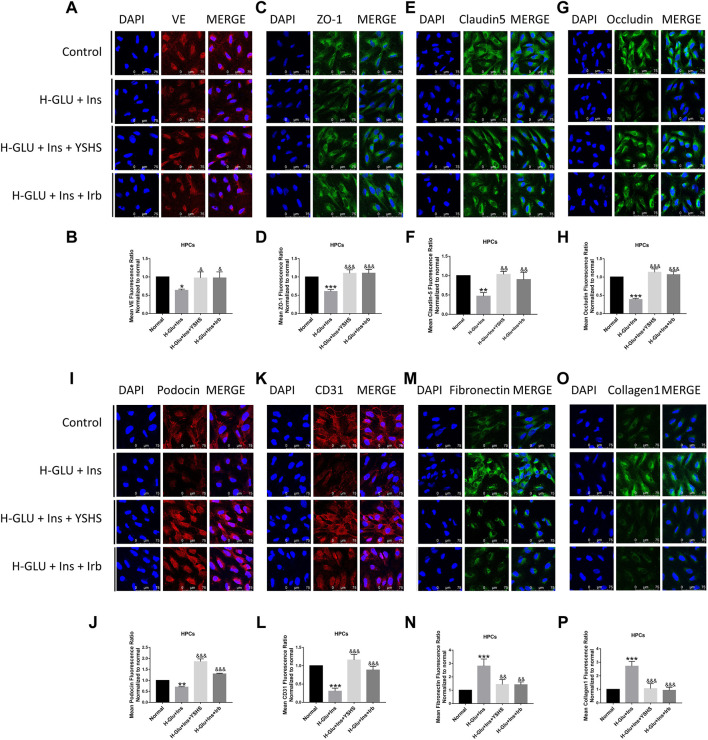
Effect of YSHS on the glomerular filtration barrier in HPC cells. Immonofluorescence assay for HPC tight junction protein VE **(A)**, ZO-1 **(C)**, claudin-5 **(E)**, and occludin **(G)** under a laser scanning confocal microscope; and relative fluorescence intensity for them **(B,D,F,G)** were determined. YSHS increased podocin **(I)** and CD31 **(K)** expressions, and relative fluorescence intensity for them **(J,L)** were determined. YSHS decreased fibronectin **(M)** and collagen1 **(O)** expressions, and relative fluorescence intensity for them **(N,P)** were determined. Values are presented as means ± SEM. *p < 0.05, **p < 0.01, ***p < 0.001, vs. control; ^&^ *p* < 0.05, ^&&^ *p* < 0.01, ^&&&^ *p* < 0.001, vs. H-Glu + Ins.

Integrity of the glomerular filtration barrier plays a central role against proteinuria. In the present study, we found (Figures 9A–H, Figures 10A–H) high glucose and high insulin significantly inhibited the expression of tight junction proteins including ZO-1, VE, occludin, and claudin 5 on both GEnC and HPC cells; while administration with YSHS significantly increased expression of tight junction proteins on the cells.

Studies have shown (Asfour et al., 2021; Darwish et al., 2021) that there are elevated levels of VEGF in diabetic patients and animal models of diabetes, and elevated VEGF has harmful effects on the kidneys. In our present study, as shown in Figures 9I–L, YSHS treatment significantly reduced expression of VEGF and VEGFR1 in GEnC cells.

Podocin and CD31 are two factors that mediate and preserve development and function of the filtration barrier. We found their expression was significantly inhibited in HPC cells treated with high glucose and insulin, and this was reversed by YSHS administration (Figures 10I–L).

The glomerular basement membrane (GBM) is a gel-like network structure composed of cell adhesion glycoproteins. Fibronectin and collagen are two essential components of the extracellular matrix that plays an important role in keeping the function of GBM. We found their expression was significantly elevated in high glucose and insulin settings, and YSHS treatment reduced their expression to the normal levels (Figures 9M–P, Figures 10M–P).

## 4 Discussion

Diabetic nephropathy is one of a serious chronic complication of diabetes, and 20%–40% of the DN population can develop end-stage renal disease (van der Sande et al., 2016). Conventional strategies, such as renin–angiotensin–aldosterone system blockade, blood glucose level control, and body-weight reduction, may not achieve satisfactory outcomes in many clinical practices. Application of Chinese medicine for DN has shown good results and has become increasingly recognized worldwide (Tang et al., 2021). In the present study, we demonstrated effects and mechanism of a Chinese prescription medicine YSHS on inhibiting the progression of DN.

YSHS granule is a traditional Chinese Medicine prescription used in the clinic for treatment of renal disease. It is mainly composed of *Ginseng* (*Ren-Shen* in Chinese), *Astragali Radix* (*Huang-Qi* in Chinese), *Atractylodes macrocephala* (*Bai-Zhu* in Chinese), and *Poria cocos* (*Fu-Ling* in Chinese). In the year 2020, an expert consensus from China Association of Chinese Medicine (The Panel of Yishen Huashi in China Association of Chinese Medicine, 2020) indicated that YSHS has definite effects on reducing proteinuria and ameliorating kidney dysfunction in both experimental and clinical study, but the underlying mechanism is still not understood. In the present study, we found that YSHS administration significantly reduced the levels of BUN, Cr, and mALB; moreover, by TEM and H&E observation, we found that the structure of both the glomerulus and filtration barrier was preserved by YSHS.

Proteinuria is the main manifestation of DN (Garsen et al., 2016), and elevated level of microalbumin (mALB) is found to be the initial characteristic of DN (Zhang et al., 2017; Uçaktürk et al., 2019). Although the underlying mechanism is not fully understood yet, alteration of the structure and function of the glomerular filtration membrane is believed to be the central event. The glomerular filtration membrane is mainly composed of glomerular endothelar endothelial cells and podocytes; due to special circumstances, these two kinds of cells are easily damaged. It is found that the elevated level of blood glucose would alter hemodynamics of the kidney in the early stage of DN (Zakiyanov et al., 2021), and this will increase the pressure in the glomerulus (Fan et al., 2021) and consequently raise the glomerular filtration rate. When this state lasts long, it can damage endothelial cells, thicken the basement membrane, and thus form the pathologic basis of DN (Marshall, 2016). Based on this pathophysiological alteration, the RAS antagonist is applied as first-line drugs for treatment of DN in the clinic. In the present study, although we did not find significant effects of YSHS on reducing blood glucose in DN rats, an obvious reduction of HbA1c was observed. As HbA1c represents a long-term level of blood glucose, our present finding obviously suggests benefit effects of YSHS on stabilizing blood glucose.

Oxidative stress plays a significant role in the development of DN. The kidney is one of the organs that are more sensitive to oxidative damage (Jha et al., 2016; Wang et al., 2020). It is found that oxidative stress will accelerate the occurrence and development of DN through a variety of pathways (Jiao et al., 2016), including increase in glomerular hyperfiltration and directly induce renal cell damage. In the present study, we found YSHS can effectively ameliorate the oxidative stress both *in vivo* and *in vitro*. On application of YSHS, generation of MDA was significantly inhibited, while levels of both SOD and GST-Pi were increased. In addition to that, we found YSHS significantly preserved viability of both glomerular endothelial cells and podocytes induced by high glucose and high insulin; moreover, expression of tight junction proteins was also found to be upregulated in these cells. Our present study demonstrated that YSHS ameliorated development of DN via preserving integrity of the glomerular filtration barrier.

Cross-talk between glomerular endothelial cells and podocytes plays an important role in maintaining function of the kidney. In the glomerulus, VEGF released from podocytes plays a key role in promoting endothelial fenestration integrity and regulating barrier function (Hayman et al., 2012; Enuwosa et al., 2021). However, excessive VEGF release may promote development of DN (Kim et al., 2005). In both DN patients and animal models, the VEGF level was found to be positively correlated with severity of albuminuria (Shao et al., 2016; Li et al., 2017). In our present study, we found high glucose and high insulin stimulation significantly increased the expression of VEGF and VEGFR1 in GEnCs cells, and YSHS intervention dramatically decreased their expression. Existing research (Wang et al., 2019) confirms that VEGF plays an important role in the production of early diabetic proteinuria. Our present findings suggest that modulating the expression of VEGF and VEGFR1 might be a key mechanism of YSHS on ameliorating DN.

Renal fibrosis is an irreversable process during development of DN (Bhattacharjee et al., 2016), and restrain fibrosis is believed to be a pivotal strategy in treatment of the disease. Chronic accumulation of extracellular matrix (ECM) will lead to fibrosis. During this process, fibronectin is believed to be key modulator. Fibronectin is a matrix glycoprotein that can be secreted by many cell types (Bowers et al., 2019). It is found that fibronectin appears in the early stage of fibrosis, and its persistent over-expression is positively correlated with renal fibrosis (Hocking et al., 2000). Inhibition of fibronectin was found to be beneficial in both ischemia-reperfusion-induced kidney disease (Bowers et al., 2019) and heart failure (Valiente-Alandi et al., 2018). Therefore, fibronectin is believed to be a potential therapeutic target of DN. In the present study, we found YSHS administration significantly reduced expression of fibronectin in the kidney tissue of DN animals, suggesting YSHS has positive effects on inhibiting renal fibrosis in the early stage of DN. This was further verified in both GEnC and HPC cells that YSHS reduced expression of both fibronectin and collagen 1.

Previous studies (Kramer-Zucker et al., 2005; Chen et al., 2021) have found that the decrease or disappearance of the expression of Nephrin/Podoein protein in the glomerulus makes the structure of the slit membrane abnormal and unable to form a normal podocyte foot. It indicates that podocin plays an important role in maintaining the integrity of the podocyte structure and slit diaphragm and was a potential target to reduce generation of proteinuria. Both animal experiments and cell experiments in this study verified that YSHS has a protective effect on upregulating podocin protein expression.

Signaling pathways involved in glucose metabolism regulation, antioxidation, anti-inflammation, anti-fibrosis, and podocyte protection have been identified as crucial mechanisms on inhibiting the progression of DN. The PI3K/AKT signaling pathway is vital for cell proliferation, growth, and viability (Jing et al., 2017). The results of network pharmacology show that the PI3K/AKT/mTOR signaling pathway has an effect on ameliorating DN by replenishing kidney and removing dampness. The results of metabolite analysis of the urine show that there were 287 specific metabolic biomarkers that distinguished among the YSHS, DN, and normal groups, and a total of 171 pathways were involved, and the genes were binned into diabetic nephropathy pathways like the glucagon signaling pathway, insulin secretion, insulin resistance, insulin signaling pathway, etc. There are some differences between the pathway analyzed by metabolomic study and the pathway analyzed by network pharmacology, but there are some hints that the changes are related to insulin resistance, and PI3K and Akt are key insulin signaling molecules in the body, which have significant regulatory effects on glucose and DN progression (Cui et al., 2019). It was verified in the present study that YSHS significantly inhibited the phosphorylation of PI3K/AKT/mTOR, thereby delaying the progression of DN.

**FIGURE 11:**
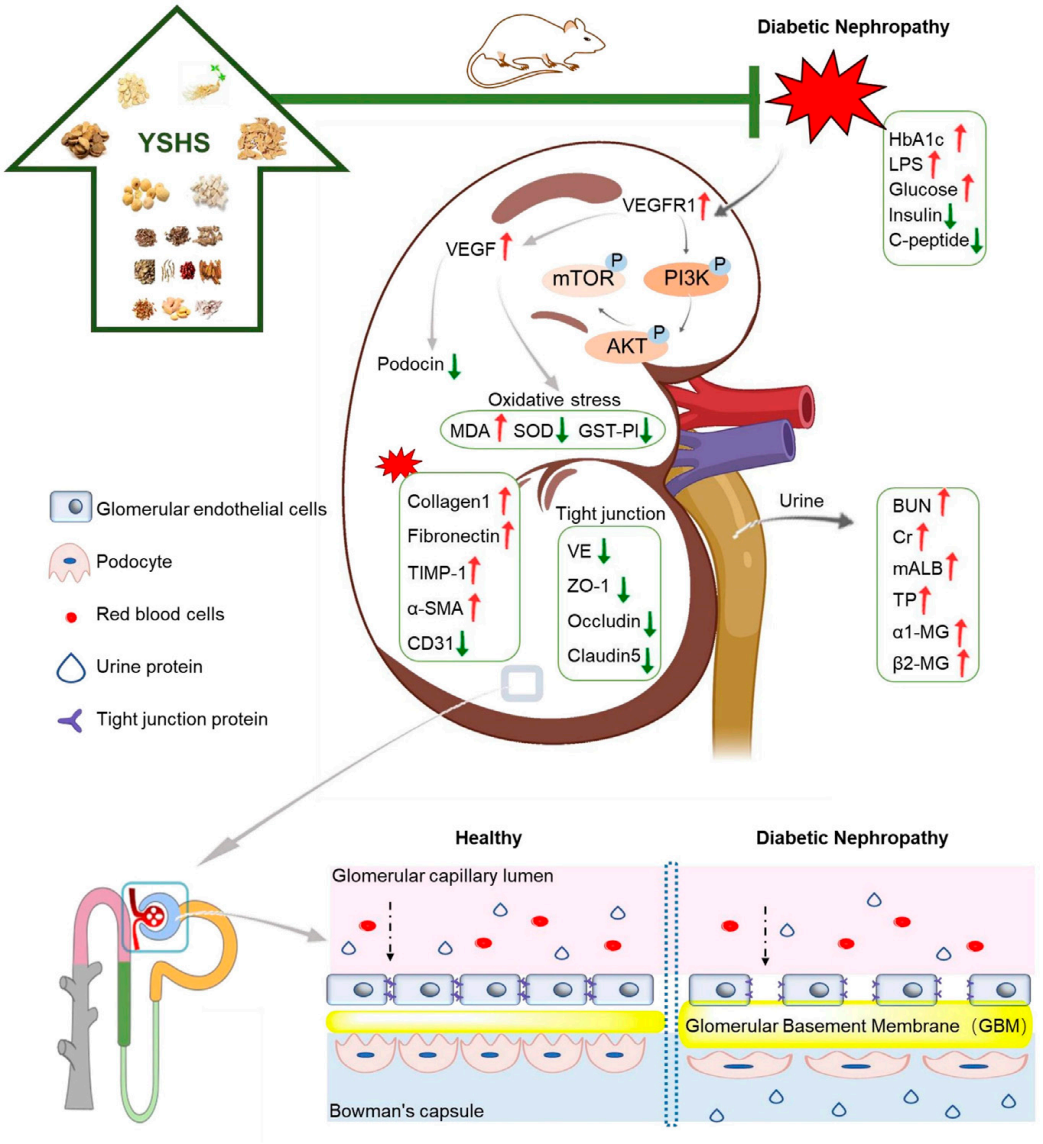
Proposed mechanism of YSHS on protecting the glomerular filtration barrier under DN settings.

In conclusion, we found in the present study that YSHS inhibited progression of DN *via* ameliorating renal fibrosis and preserving the integrity of the kidney filtration barrier, and the PI3K/AKT/mTOR pathway played a pivotal role in it (Figure 11). Our present findings provided pharmacological evidence of YSHS on kidney diseases.

## Data Availability

The original contributions presented in the study are included in the article/Supplementary Material; further inquiries can be directed to the corresponding authors.
