# Supplementary material for: Yishen Huashi Granules Ameliorated the Development of Diabetic Nephropathy by Reducing the Damage of Glomerular Filtration Barrier
Source: Front Pharmacol. 2022 Jul 22;13:872940. doi: 10.3389/fphar.2022.872940 (PMC9353776; doi:10.3389/fphar.2022.872940)
Supplement: Supplementary file 1 [file Table1.DOCX]

**Supplementary Table 1.** Potentially active compounds derived from network pharmacological analysis.

| Renshen (*Panax Ginseng C. A. Mey.*) | | | | |
| --- | --- | --- | --- | --- |
| **Mol ID** | **Molecule Name** | **MW** | **OB (%)** | **DL** |
| MOL002879 | Diop | 390.62 | 43.59 | 0.39 |
| MOL000449 | Stigmasterol | 412.77 | 43.83 | 0.76 |
| MOL000358 | beta-sitosterol | 414.79 | 36.91 | 0.75 |
| MOL003648 | Inermin | 284.28 | 65.83 | 0.54 |
| MOL000422 | kaempferol | 286.25 | 41.88 | 0.24 |
| MOL004492 | Chrysanthemaxanthin | 584.96 | 38.72 | 0.58 |
| MOL005308 | Aposiopolamine | 271.34 | 66.65 | 0.22 |
| MOL005314 | Celabenzine | 379.55 | 101.88 | 0.49 |
| MOL005317 | Deoxyharringtonine | 515.66 | 39.27 | 0.81 |
| MOL005318 | Dianthramine | 289.26 | 40.45 | 0.2 |
| MOL005320 | arachidonate | 304.52 | 45.57 | 0.2 |
| MOL005321 | Frutinone A | 264.24 | 65.9 | 0.34 |
| MOL005344 | ginsenoside rh2 | 622.98 | 36.32 | 0.56 |
| MOL005348 | Ginsenoside-Rh4_qt | 458.8 | 31.11 | 0.78 |
| MOL005356 | Girinimbin | 263.36 | 61.22 | 0.31 |
| MOL005357 | Gomisin B | 514.62 | 31.99 | 0.83 |
| MOL005360 | malkangunin | 432.56 | 57.71 | 0.63 |
| MOL005376 | Panaxadiol | 460.82 | 33.09 | 0.79 |
| MOL005384 | suchilactone | 368.41 | 57.52 | 0.56 |
| MOL005399 | alexandrin_qt | 414.79 | 36.91 | 0.75 |
| MOL005401 | ginsenoside Rg5_qt | 442.8 | 39.56 | 0.79 |
| MOL000787 | Fumarine | 353.4 | 59.26 | 0.83 |
|  |  |  |  |  |
| Huangqi (*Hedysarum Multijugum Maxim*) | | | | |
| **Mol ID** | **Molecule Name** | **MW** | **OB (%)** | **DL** |
| MOL000211 | Mairin | 456.78 | 55.38 | 0.78 |
| MOL000239 | Jaranol | 314.31 | 50.83 | 0.29 |
| MOL000296 | hederagenin | 414.79 | 36.91 | 0.75 |
| MOL000033 | (3S,8S,9S,10R,13R,14S,17R)-10,13-dimethyl-17-[(2R,5S)-5-propan-2-yloctan-2-yl]-2,3,4,7,8,9,11,12,14,15,16,17-dodecahydro-1H-cyclopenta[a]phenanthren-3-ol | 428.82 | 36.23 | 0.78 |
| MOL000354 | isorhamnetin | 316.28 | 49.6 | 0.31 |
| MOL000371 | 3,9-di-O-methylnissolin | 314.36 | 53.74 | 0.48 |
| MOL000374 | 5'-hydroxyiso-muronulatol-2',5'-di-O-glucoside | 642.67 | 41.72 | 0.69 |
| MOL000378 | 7-O-methylisomucronulatol | 316.38 | 74.69 | 0.3 |
| MOL000379 | 9,10-dimethoxypterocarpan-3-O-β-D-glucoside | 462.49 | 36.74 | 0.92 |
| MOL000380 | (6aR,11aR)-9,10-dimethoxy-6a,11a-dihydro-6H-benzofurano[3,2-c]chromen-3-ol | 300.33 | 64.26 | 0.42 |
| MOL000387 | Bifendate | 418.38 | 31.1 | 0.67 |
| MOL000392 | formononetin | 268.28 | 69.67 | 0.21 |
| MOL000398 | isoflavanone | 316.33 | 109.99 | 0.3 |
| MOL000417 | Calycosin | 284.28 | 47.75 | 0.24 |
| MOL000422 | kaempferol | 286.25 | 41.88 | 0.24 |
| MOL000433 | FA | 441.45 | 68.96 | 0.71 |
| MOL000438 | (3R)-3-(2-hydroxy-3,4-dimethoxyphenyl)chroman-7-ol | 302.35 | 67.67 | 0.26 |
| MOL000439 | isomucronulatol-7,2'-di-O-glucosiole | 626.67 | 49.28 | 0.62 |
| MOL000442 | 1,7-Dihydroxy-3,9-dimethoxy pterocarpene | 314.31 | 39.05 | 0.48 |
| MOL000098 | quercetin | 302.25 | 46.43 | 0.28 |
|  |  |  |  |  |
| Baizhu (*Atractylodes Macrocephala Koidz*) | | | | |
| **Mol ID** | **Molecule Name** | **MW** | **OB (%)** | **DL** |
| MOL000072 | 8β-ethoxy atractylenolide Ⅲ | 276.41 | 35.95 | 0.21 |
| MOL000033 | (3S,8S,9S,10R,13R,14S,17R)-10,13-dimethyl-17-[(2R,5S)-5-propan-2-yloctan-2-yl]-2,3,4,7,8,9,11,12,14,15,16,17-dodecahydro-1H-cyclopenta[a]phenanthren-3-ol | 428.82 | 36.23 | 0.78 |
| MOL000028 | α-Amyrin | 426.8 | 39.51 | 0.76 |
| MOL000049 | 3β-acetoxyatractylone | 274.39 | 54.07 | 0.22 |
| MOL000021 | 14-acetyl-12-senecioyl-2E,8E,10E-atractylentriol | 355.44 | 60.31 | 0.31 |
| MOL000020 | 12-senecioyl-2E,8E,10E-atractylentriol | 312.39 | 62.4 | 0.22 |
| MOL000022 | 14-acetyl-12-senecioyl-2E,8Z,10E-atractylentriol | 356.45 | 63.37 | 0.3 |
|  |  |  |  |  |
| Fuling （*Poria Cocos(Schw.) Wolf.*） | | | | |
| **Mol ID** | **Molecule Name** | **MW** | **OB (%)** | **DL** |
| MOL000273 | (2R)-2-[(3S,5R,10S,13R,14R,16R,17R)-3,16-dihydroxy-4,4,10,13,14-pentamethyl-2,3,5,6,12,15,16,17-octahydro-1H-cyclopenta[a]phenanthren-17-yl]-6-methylhept-5-enoic acid | 470.76 | 30.93 | 0.81 |
| MOL000275 | trametenolic acid | 456.78 | 38.71 | 0.8 |
| MOL000276 | 7,9(11)-dehydropachymic acid | 526.83 | 35.11 | 0.81 |
| MOL000279 | Cerevisterol | 430.74 | 37.96 | 0.77 |
| MOL000280 | (2R)-2-[(3S,5R,10S,13R,14R,16R,17R)-3,16-dihydroxy-4,4,10,13,14-pentamethyl-2,3,5,6,12,15,16,17-octahydro-1H-cyclopenta[a]phenanthren-17-yl]-5-isopropyl-hex-5-enoic acid | 484.79 | 31.07 | 0.82 |
| MOL000282 | ergosta-7,22E-dien-3beta-ol | 398.74 | 43.51 | 0.72 |
| MOL000283 | Ergosterol peroxide | 430.74 | 40.36 | 0.81 |
| MOL000285 | (2R)-2-[(5R,10S,13R,14R,16R,17R)-16-hydroxy-3-keto-4,4,10,13,14-pentamethyl-1,2,5,6,12,15,16,17-octahydrocyclopenta[a]phenanthren-17-yl]-5-isopropyl-hex-5-enoic acid | 482.77 | 38.26 | 0.82 |
| MOL000287 | 3beta-Hydroxy-24-methylene-8-lanostene-21-oic acid | 470.81 | 38.7 | 0.81 |
| MOL000289 | pachymic acid | 528.85 | 33.63 | 0.81 |
| MOL000290 | Poricoic acid A | 498.77 | 30.61 | 0.76 |
| MOL000291 | Poricoic acid B | 484.74 | 30.52 | 0.75 |
| MOL000292 | poricoic acid C | 482.77 | 38.15 | 0.75 |
| MOL000296 | hederagenin | 414.79 | 36.91 | 0.75 |
| MOL000300 | dehydroeburicoic acid | 453.75 | 44.17 | 0.83 |
|  |  |  |  |  |
| Banxia (*Arum Ternatum Thunb*) | | | | |
| **Mol ID** | **Molecule Name** | **MW** | **OB (%)** | **DL** |
| MOL002670 | Cavidine | 353.45 | 35.64 | 0.81 |
| MOL003578 | Cycloartenol | 426.8 | 38.69 | 0.78 |
| MOL005030 | gondoic acid | 310.58 | 30.7 | 0.2 |
| MOL000449 | Stigmasterol | 412.77 | 43.83 | 0.76 |
| MOL006936 | 10,13-eicosadienoic | 308.56 | 39.99 | 0.2 |
| MOL000358 | beta-sitosterol | 414.79 | 36.91 | 0.75 |
| MOL006937 | 12,13-epoxy-9-hydroxynonadeca-7,10-dienoic acid | 324.51 | 42.15 | 0.24 |
| MOL001755 | 24-Ethylcholest-4-en-3-one | 412.77 | 36.08 | 0.76 |
| MOL000519 | coniferin | 314.41 | 31.11 | 0.32 |
| MOL006967 | beta-D-Ribofuranoside, xanthine-9 | 284.26 | 44.72 | 0.21 |
| MOL002714 | baicalein | 270.25 | 33.52 | 0.21 |
| MOL002776 | Baicalin | 446.39 | 40.12 | 0.75 |
| MOL006957 | (3S,6S)-3-(benzyl)-6-(4-hydroxybenzyl)piperazine-2,5-quinone | 310.38 | 46.89 | 0.27 |
|  |  |  |  |  |
| Zexie (*Alisma Orientale (Sam.) Juz*) | | | | |
| **Mol ID** | **Molecule Name** | **MW** | **OB (%)** | **DL** |
| MOL000359 | sitosterol | 414.79 | 36.91 | 0.75 |
| MOL000830 | Alisol B | 472.78 | 34.47 | 0.82 |
| MOL000831 | Alisol B monoacetate | 514.82 | 35.58 | 0.81 |
| MOL000832 | alisol,b,23-acetate | 446.74 | 32.52 | 0.82 |
| MOL000849 | 16β-methoxyalisol B monoacetate | 544.85 | 32.43 | 0.77 |
| MOL000853 | alisol B | 444.72 | 36.76 | 0.82 |
| MOL000854 | alisol C | 486.76 | 32.7 | 0.82 |
| MOL000856 | alisol C monoacetate | 514.77 | 33.06 | 0.83 |
| MOL002464 | 1-Monolinolein | 354.59 | 37.18 | 0.3 |
| MOL000862 | [(1S,3R)-1-[(2R)-3,3-dimethyloxiran-2-yl]-3-[(5R,8S,9S,10S,11S,14R)-11-hydroxy-4,4,8,10,14-pentamethyl-3-oxo-1,2,5,6,7,9,11,12,15,16-decahydrocyclopenta[a]phenanthren-17-yl]butyl] acetate | 514.82 | 35.58 | 0.81 |
