# Supplementary material for: Yishen Huashi Granules Ameliorated the Development of Diabetic Nephropathy by Reducing the Damage of Glomerular Filtration Barrier
Source: Front Pharmacol. 2022 Jul 22;13:872940. doi: 10.3389/fphar.2022.872940 (PMC9353776; doi:10.3389/fphar.2022.872940)
Supplement: Supplementary file 2 [file Table2.DOCX]

**Supplementary Table 2.** Possible pathways identified by urine metabolite analysis.

|  | **Total** | **Hits** | **Raw p** | **-log(p)** | **Impact** | **pathway** |
| --- | --- | --- | --- | --- | --- | --- |
| ABC transporters | 138 | 26 | 8.87E-05 | 9.3308 | 0.18841 | rno02010 |
| Phenylalanine metabolism | 60 | 14 | 0.000432 | 7.7462 | 0.35714 | rno00360 |
| Central carbon metabolism in cancer | 37 | 14 | 9.55E-07 | 13.861 | 0.39623 | rno05230 |
| Arginine and proline metabolism | 78 | 13 | 0.015026 | 4.198 | 0.18873 | rno00330 |
| Bile secretion | 97 | 11 | 0.21459 | 1.539 | 0.10092 | rno04976 |
| Protein digestion and absorption | 47 | 11 | 0.001733 | 6.3582 | 0.23404 | rno04974 |
| Tyrosine metabolism | 78 | 10 | 0.13304 | 2.0171 | 0.088 | rno00350 |
| Pyrimidine metabolism | 65 | 10 | 0.05044 | 2.987 | 0.17536 | rno00240 |
| Nicotinate and nicotinamide metabolism | 55 | 10 | 0.017705 | 4.0339 | 0.40465 | rno00760 |
| Histidine metabolism | 47 | 10 | 0.005812 | 5.1479 | 0.416 | rno00340 |
| Cysteine and methionine metabolism | 63 | 9 | 0.090133 | 2.4065 | 0.26371 | rno00270 |
| Pentose and glucuronate interconversions | 56 | 9 | 0.048776 | 3.0205 | 0.2931 | rno00040 |
| Glycine, serine and threonine metabolism | 50 | 9 | 0.025403 | 3.6729 | 0.18323 | rno00260 |
| beta-Alanine metabolism | 32 | 9 | 0.001132 | 6.7838 | 0.46552 | rno00410 |
| Purine metabolism | 95 | 8 | 0.58785 | 0.53128 | 0.13667 | rno00230 |
| Glyoxylate and dicarboxylate metabolism | 62 | 8 | 0.16353 | 1.8107 | 0.2 | rno00630 |
| Ascorbate and aldarate metabolism | 50 | 8 | 0.06271 | 2.7692 | 0.38961 | rno00053 |
| Taste transduction | 32 | 8 | 0.004763 | 5.3469 | 0.26316 | rno04742 |
| Pantothenate and CoA biosynthesis | 30 | 8 | 0.003087 | 5.7805 | 0.27679 | rno00770 |
| Tryptophan metabolism | 83 | 7 | 0.58753 | 0.53183 | 0.063444 | rno00380 |
| Aminoacyl-tRNA biosynthesis | 52 | 7 | 0.15865 | 1.8411 | 0.125 | rno00970 |
| Alanine, aspartate and glutamate metabolism | 28 | 7 | 0.008173 | 4.807 | 0.53371 | rno00250 |
| Glucagon signaling pathway | 26 | 7 | 0.005276 | 5.2446 | 0.27273 | rno04922 |
| Neuroactive ligand-receptor interaction | 52 | 6 | 0.29251 | 1.2293 | 0.11538 | rno04080 |
| Lysine degradation | 50 | 6 | 0.26182 | 1.3401 | 0.2013 | rno00310 |
| Propanoate metabolism | 48 | 6 | 0.23206 | 1.4608 | 0.12308 | rno00640 |
| Galactose metabolism | 46 | 6 | 0.20347 | 1.5922 | 0.079365 | rno00052 |
| Vitamin digestion and absorption | 39 | 6 | 0.11587 | 2.1553 | 0.14286 | rno04977 |
| Pyruvate metabolism | 31 | 6 | 0.04637 | 3.0711 | 0.18367 | rno00620 |
| Linoleic acid metabolism | 28 | 6 | 0.029494 | 3.5236 | 0.23684 | rno00591 |
| Valine, leucine and isoleucine biosynthesis | 23 | 6 | 0.011393 | 4.4748 | 0.25806 | rno00290 |
| Butanoate metabolism | 42 | 5 | 0.29654 | 1.2156 | 0.29412 | rno00650 |
| Arginine biosynthesis | 23 | 5 | 0.043168 | 3.1427 | 0.31447 | rno00220 |
| Taurine and hypotaurine metabolism | 22 | 5 | 0.036261 | 3.317 | 0.26471 | rno00430 |
| Citrate cycle (TCA cycle) | 20 | 5 | 0.024607 | 3.7047 | 0.2 | rno00020 |
| Fructose and mannose metabolism | 54 | 4 | 0.70057 | 0.35586 | 0.079365 | rno00051 |
| Inositol phosphate metabolism | 47 | 4 | 0.59064 | 0.52654 | 0.049881 | rno00562 |
| Starch and sucrose metabolism | 37 | 4 | 0.40032 | 0.91548 | 0.22314 | rno00500 |
| Pentose phosphate pathway | 35 | 4 | 0.35962 | 1.0227 | 0.087179 | rno00030 |
| Inflammatory mediator regulation of TRP channels | 35 | 4 | 0.35962 | 1.0227 | 0.15385 | rno04750 |
| Sulfur metabolism | 33 | 4 | 0.31886 | 1.143 | 0.072727 | rno00920 |
| Mineral absorption | 29 | 4 | 0.23913 | 1.4308 | 0.11429 | rno04978 |
| Ferroptosis | 29 | 4 | 0.23913 | 1.4308 | 0.18605 | rno04216 |
| Carbohydrate digestion and absorption | 27 | 4 | 0.20121 | 1.6034 | 0.17241 | rno04973 |
| cAMP signaling pathway | 25 | 4 | 0.16535 | 1.7997 | 0.18519 | rno04024 |
| Insulin resistance | 20 | 4 | 0.08832 | 2.4268 | 0.20833 | rno04931 |
| Insulin secretion | 12 | 4 | 0.015573 | 4.1622 | 0.35 | rno04911 |
| GABAergic synapse | 9 | 4 | 0.004884 | 5.3219 | 0.52941 | rno04727 |
| Ubiquinone and other terpenoid-quinone biosynthesis | 92 | 3 | 0.98938 | 0.010678 | 0.05625 | rno00130 |
| Fatty acid biosynthesis | 58 | 3 | 0.89034 | 0.11615 | 0.020076 | rno00061 |
| Folate biosynthesis | 58 | 3 | 0.89034 | 0.11615 | 0.024038 | rno00790 |
| alpha-Linolenic acid metabolism | 44 | 3 | 0.74762 | 0.29086 | 0.045455 | rno00592 |
| Serotonergic synapse | 42 | 3 | 0.71826 | 0.33093 | 0.16129 | rno04726 |
| Glutathione metabolism | 38 | 3 | 0.65178 | 0.42804 | 0.11189 | rno00480 |
| Phenylalanine, tyrosine and tryptophan biosynthesis | 34 | 3 | 0.57489 | 0.55358 | 0.13158 | rno00400 |
| Pathways of neurodegeneration - multiple diseases | 32 | 3 | 0.53271 | 0.62978 | 0.0625 | rno05022 |
| Glycolysis / Gluconeogenesis | 31 | 3 | 0.51076 | 0.67186 | 0.070539 | rno00010 |
| Vitamin B6 metabolism | 29 | 3 | 0.46532 | 0.76502 | 0.14925 | rno00750 |
| Retrograde endocannabinoid signaling | 19 | 3 | 0.22346 | 1.4985 | 0.15789 | rno04723 |
| Proximal tubule bicarbonate reclamation | 17 | 3 | 0.1774 | 1.7293 | 0.20354 | rno04964 |
| HIF-1 signaling pathway | 15 | 3 | 0.13448 | 2.0064 | 0.31579 | rno04066 |
| Gastric acid secretion | 14 | 3 | 0.11457 | 2.1666 | 0.10714 | rno04971 |
| Amoebiasis | 13 | 3 | 0.095897 | 2.3445 | 0.23077 | rno05146 |
| Synaptic vesicle cycle | 12 | 3 | 0.078612 | 2.5432 | 0.25 | rno04721 |
| Amino sugar and nucleotide sugar metabolism | 108 | 2 | 0.99945 | 0.000555 | 0.006897 | rno00520 |
| Neomycin, kanamycin and gentamicin biosynthesis | 81 | 2 | 0.99472 | 0.005289 | 0.036145 | rno00524 |
| Biosynthesis of unsaturated fatty acids | 74 | 2 | 0.99069 | 0.009351 | 0.041667 | rno01040 |
| Primary bile acid biosynthesis | 47 | 2 | 0.9241 | 0.078938 | 0.078049 | rno00120 |
| Valine, leucine and isoleucine degradation | 42 | 2 | 0.89048 | 0.11599 | 0.079051 | rno00280 |
| Thiamine metabolism | 31 | 2 | 0.76365 | 0.26965 | 0.088889 | rno00730 |
| Pathways in cancer | 31 | 2 | 0.76365 | 0.26965 | 0.090909 | rno05200 |
| Biotin metabolism | 28 | 2 | 0.7118 | 0.33995 | 0.15625 | rno00780 |
| Ovarian steroidogenesis | 24 | 2 | 0.62831 | 0.46471 | 0.090909 | rno04913 |
| Thermogenesis | 23 | 2 | 0.60472 | 0.50299 | 0.10256 | rno04714 |
| AMPK signaling pathway | 22 | 2 | 0.58001 | 0.54471 | 0.066667 | rno04152 |
| Aldosterone synthesis and secretion | 22 | 2 | 0.58001 | 0.54471 | 0.075 | rno04925 |
| Thyroid hormone synthesis | 21 | 2 | 0.55419 | 0.59025 | 0.028986 | rno04918 |
| Salivary secretion | 17 | 2 | 0.44023 | 0.82047 | 0.10526 | rno04970 |
| Vascular smooth muscle contraction | 16 | 2 | 0.40931 | 0.89329 | 0.125 | rno04270 |
| Pancreatic secretion | 15 | 2 | 0.37758 | 0.97398 | 0.051282 | rno04972 |
| Regulation of lipolysis in adipocytes | 14 | 2 | 0.34516 | 1.0637 | 0.10714 | rno04923 |
| Amyotrophic lateral sclerosis | 14 | 2 | 0.34516 | 1.0637 | 0.11111 | rno05014 |
| Platelet activation | 14 | 2 | 0.34516 | 1.0637 | 0.14286 | rno04611 |
| D-Glutamine and D-glutamate metabolism | 13 | 2 | 0.31222 | 1.164 | 0.2381 | rno00471 |
| Oxytocin signaling pathway | 12 | 2 | 0.27894 | 1.2768 | 0.16667 | rno04921 |
| Cholinergic synapse | 12 | 2 | 0.27894 | 1.2768 | 0.2 | rno04725 |
| Phospholipase D signaling pathway | 11 | 2 | 0.24555 | 1.4042 | 0.095238 | rno04072 |
| Gap junction | 11 | 2 | 0.24555 | 1.4042 | 0.18182 | rno04540 |
| Fc epsilon RI signaling pathway | 11 | 2 | 0.24555 | 1.4042 | 0.18182 | rno04664 |
| Alcoholism | 10 | 2 | 0.21233 | 1.5496 | 0.14286 | rno05034 |
| Pertussis | 10 | 2 | 0.21233 | 1.5496 | 0.2 | rno05133 |
| Amphetamine addiction | 9 | 2 | 0.17962 | 1.7169 | 0.15385 | rno05031 |
| Circadian entrainment | 9 | 2 | 0.17962 | 1.7169 | 0.22222 | rno04713 |
| Long-term depression | 9 | 2 | 0.17962 | 1.7169 | 0.22222 | rno04730 |
| Longevity regulating pathway | 8 | 2 | 0.14779 | 1.912 | 0.25 | rno04211 |
| Glutamatergic synapse | 8 | 2 | 0.14779 | 1.912 | 0.3 | rno04724 |
| Cocaine addiction | 7 | 2 | 0.11732 | 2.1429 | 0.18182 | rno05030 |
| Nicotine addiction | 7 | 2 | 0.11732 | 2.1429 | 0.28571 | rno05033 |
| Long-term potentiation | 7 | 2 | 0.11732 | 2.1429 | 0.28571 | rno04720 |
| Chagas disease | 6 | 2 | 0.088733 | 2.4221 | 0.33333 | rno05142 |
| GnRH signaling pathway | 6 | 2 | 0.088733 | 2.4221 | 0.33333 | rno04912 |
| Porphyrin and chlorophyll metabolism | 142 | 1 | 1 | 1.94E-06 | 0.003922 | rno00860 |
| Chemical carcinogenesis | 99 | 1 | 0.99989 | 0.000111 | 0.007194 | rno05204 |
| Steroid hormone biosynthesis | 99 | 1 | 0.99989 | 0.000111 | 0.007968 | rno00140 |
| Arachidonic acid metabolism | 75 | 1 | 0.99896 | 0.001037 | 0.16244 | rno00590 |
| Phosphonate and phosphinate metabolism | 56 | 1 | 0.994 | 0.00602 | 0.014706 | rno00440 |
| Glycerophospholipid metabolism | 52 | 1 | 0.99132 | 0.008714 | 0.007407 | rno00564 |
| Drug metabolism - other enzymes | 52 | 1 | 0.99132 | 0.008714 | 0.008876 | rno00983 |
| Fatty acid degradation | 50 | 1 | 0.98957 | 0.010484 | 0.00303 | rno00071 |
| Terpenoid backbone biosynthesis | 45 | 1 | 0.98349 | 0.016652 | 0.006536 | rno00900 |
| Glycerolipid metabolism | 38 | 1 | 0.96861 | 0.03189 | 0.012987 | rno00561 |
| Phosphatidylinositol signaling system | 29 | 1 | 0.92848 | 0.074206 | 0.035115 | rno04070 |
| Selenocompound metabolism | 27 | 1 | 0.91414 | 0.089767 | 0.072072 | rno00450 |
| Caffeine metabolism | 22 | 1 | 0.86451 | 0.14559 | 0.029412 | rno00232 |
| Parkinson disease | 21 | 1 | 0.85158 | 0.16066 | 0.090909 | rno05012 |
| Riboflavin metabolism | 20 | 1 | 0.83742 | 0.17743 | 0.19048 | rno00740 |
| Nitrogen metabolism | 19 | 1 | 0.82191 | 0.19613 | 0.090909 | rno00910 |
| Renin secretion | 17 | 1 | 0.78633 | 0.24037 | 0.14815 | rno04924 |
| Oxidative phosphorylation | 16 | 1 | 0.76597 | 0.26661 | 0.1 | rno00190 |
| Lipoic acid metabolism | 13 | 1 | 0.69255 | 0.36737 | 0.076923 | rno00785 |
| Cushing syndrome | 13 | 1 | 0.69255 | 0.36737 | 0.10526 | rno04934 |
| Dopaminergic synapse | 12 | 1 | 0.6633 | 0.41053 | 0.045455 | rno04728 |
| Cortisol synthesis and secretion | 12 | 1 | 0.6633 | 0.41053 | 0.11111 | rno04927 |
| D-Arginine and D-ornithine metabolism | 11 | 1 | 0.63127 | 0.46003 | 0.066667 | rno00472 |
| Thyroid hormone signaling pathway | 11 | 1 | 0.63127 | 0.46003 | 0.090909 | rno04919 |
| Calcium signaling pathway | 11 | 1 | 0.63127 | 0.46003 | 0.13333 | rno04020 |
| Sulfur relay system | 11 | 1 | 0.63127 | 0.46003 | 0.13333 | rno04122 |
| Prolactin signaling pathway | 11 | 1 | 0.63127 | 0.46003 | 0.15789 | rno04917 |
| Necroptosis | 10 | 1 | 0.5962 | 0.51717 | 0.083333 | rno04217 |
| Adrenergic signaling in cardiomyocytes | 10 | 1 | 0.5962 | 0.51717 | 0.1 | rno04261 |
| cGMP-PKG signaling pathway | 10 | 1 | 0.5962 | 0.51717 | 0.14286 | rno04022 |
| Parathyroid hormone synthesis, secretion and action | 10 | 1 | 0.5962 | 0.51717 | 0.16667 | rno04928 |
| One carbon pool by folate | 9 | 1 | 0.55782 | 0.58373 | 0.067797 | rno00670 |
| Apelin signaling pathway | 9 | 1 | 0.55782 | 0.58373 | 0.11111 | rno04371 |
| Fc gamma R-mediated phagocytosis | 8 | 1 | 0.51579 | 0.66205 | 0.0625 | rno04666 |
| Aldosterone-regulated sodium reabsorption | 8 | 1 | 0.51579 | 0.66205 | 0.1 | rno04960 |
| African trypanosomiasis | 8 | 1 | 0.51579 | 0.66205 | 0.125 | rno05143 |
| Estrogen signaling pathway | 8 | 1 | 0.51579 | 0.66205 | 0.125 | rno04915 |
| Endocrine and other factor-regulated calcium reabsorption | 8 | 1 | 0.51579 | 0.66205 | 0.125 | rno04961 |
| Olfactory transduction | 8 | 1 | 0.51579 | 0.66205 | 0.16667 | rno04740 |
| Morphine addiction | 8 | 1 | 0.51579 | 0.66205 | 0.2 | rno05032 |
| Ras signaling pathway | 7 | 1 | 0.46979 | 0.75546 | 0.14286 | rno04014 |
| Spinocerebellar ataxia | 7 | 1 | 0.46979 | 0.75546 | 0.14286 | rno05017 |
| Type II diabetes mellitus | 6 | 1 | 0.41944 | 0.86884 | 0.16667 | rno04930 |
| Leishmaniasis | 6 | 1 | 0.41944 | 0.86884 | 0.16667 | rno05140 |
| Huntington disease | 6 | 1 | 0.41944 | 0.86884 | 0.16667 | rno05016 |
| Melanogenesis | 6 | 1 | 0.41944 | 0.86884 | 0.16667 | rno04916 |
| Relaxin signaling pathway | 6 | 1 | 0.41944 | 0.86884 | 0.16667 | rno04926 |
| Synthesis and degradation of ketone bodies | 6 | 1 | 0.41944 | 0.86884 | 0.31818 | rno00072 |
| Regulation of actin cytoskeleton | 5 | 1 | 0.36432 | 1.0097 | 0.14286 | rno04810 |
| MAPK signaling pathway | 5 | 1 | 0.36432 | 1.0097 | 0.2 | rno04010 |
| Rap1 signaling pathway | 5 | 1 | 0.36432 | 1.0097 | 0.2 | rno04015 |
| FoxO signaling pathway | 5 | 1 | 0.36432 | 1.0097 | 0.2 | rno04068 |
| Asthma | 5 | 1 | 0.36432 | 1.0097 | 0.2 | rno05310 |
| Human T-cell leukemia virus 1 infection | 5 | 1 | 0.36432 | 1.0097 | 0.2 | rno05166 |
| Prion disease | 5 | 1 | 0.36432 | 1.0097 | 0.2 | rno05020 |
| PPAR signaling pathway | 5 | 1 | 0.36432 | 1.0097 | 0.2 | rno03320 |
| Chemokine signaling pathway | 5 | 1 | 0.36432 | 1.0097 | 0.2 | rno04062 |
| Oocyte meiosis | 4 | 1 | 0.30399 | 1.1908 | 0.25 | rno04114 |
| mTOR signaling pathway | 4 | 1 | 0.30399 | 1.1908 | 0.25 | rno04150 |
| Insulin signaling pathway | 4 | 1 | 0.30399 | 1.1908 | 0.25 | rno04910 |
| Progesterone-mediated oocyte maturation | 4 | 1 | 0.30399 | 1.1908 | 0.25 | rno04914 |
| Growth hormone synthesis, secretion and action | 4 | 1 | 0.30399 | 1.1908 | 0.25 | rno04935 |
| Leukocyte transendothelial migration | 4 | 1 | 0.30399 | 1.1908 | 0.25 | rno04670 |
| Dilated cardiomyopathy | 3 | 1 | 0.23796 | 1.4357 | 0.33333 | rno05414 |
| Human papillomavirus infection | 3 | 1 | 0.23796 | 1.4357 | 0.33333 | rno05165 |
| Renal cell carcinoma | 3 | 1 | 0.23796 | 1.4357 | 0.4 | rno05211 |
| Longevity regulating pathway - multiple species | 2 | 1 | 0.16568 | 1.7977 | 0.5 | rno04213 |
| Circadian rhythm | 2 | 1 | 0.16568 | 1.7977 | 0.5 | rno04710 |
| Vasopressin-regulated water reabsorption | 2 | 1 | 0.16568 | 1.7977 | 0.5 | rno04962 |
| Hedgehog signaling pathway | 1 | 1 | 0.086576 | 2.4467 | 1 | rno04340 |
