# Supplementary material for: Yishen Huashi Granules Ameliorated the Development of Diabetic Nephropathy by Reducing the Damage of Glomerular Filtration Barrier
Source: Front Pharmacol. 2022 Jul 22;13:872940. doi: 10.3389/fphar.2022.872940 (PMC9353776; doi:10.3389/fphar.2022.872940)
Supplement: Supplementary file 3 [file DataSheet1.docx]

**Supplementary Methods**

**Quantitative determination of calycosin isoflavone**

**Experimental equipment and materials**

1.Equipment

Japan Shimadzu High Performance Liquid Chromatograph (Model: CBM-20A; LC-20AT Pump; SPD-M20A UV Detector; SIL-20A Autosampler; Shimadzu Lab Solutions Chromatography Workstation, Japan).

Balance (Manufacturer: Beijing Sartorius Instrument System Co., Ltd.; Model: BS224s/CB-254).

2.Reagent

Acetonitrile (chromatographic purity, manufacturer: Merck; Lot number: JA090530).

3.Other consumables

Membrane: diameter 25mm, pore diameter 0.45um; Polyether sulfone membranes; Zinteng

**Experimental methods**

1. Chromatographic conditions

Column: Pursuit 5 C18 (4.0*250mm, 5um).

Mobile phase: acetonitrile-water, elution gradient as follows:

| Time | H2O% | Acetonitrile% |
| --- | --- | --- |
| 0 | 92 | 8 |
| 5 | 92 | 8 |
| 35 | 80 | 20 |
| 100 | 25 | 75 |
| 100.1 | 92 | 8 |
| 105 | 92 | 8 |

Detection wavelength: 199nm; Flow rate: 1.0mL/min; Column temperature: off; Injection volume: 10uL

2. Solution preparation

Sample solution to be measured: Pipette gun to aspirate 100μL of beneficial nephrotic wet extract (2 g/mL), add 2.0mL of methanol to dissolve, over 0.45μm membrane, set aside.

3. Measurement method

After equilibulating the column, take 10μL each of the blank and sample solutions to be measured, and inject them into the liquid chromatograph to record the chromatogram.

**Experimental results**

Content of calycosin isoflavone in YSHS granule was calculated to be 0.022 mg/mL granule. The chromatogram is shown in the Supplementary Fig. 1.


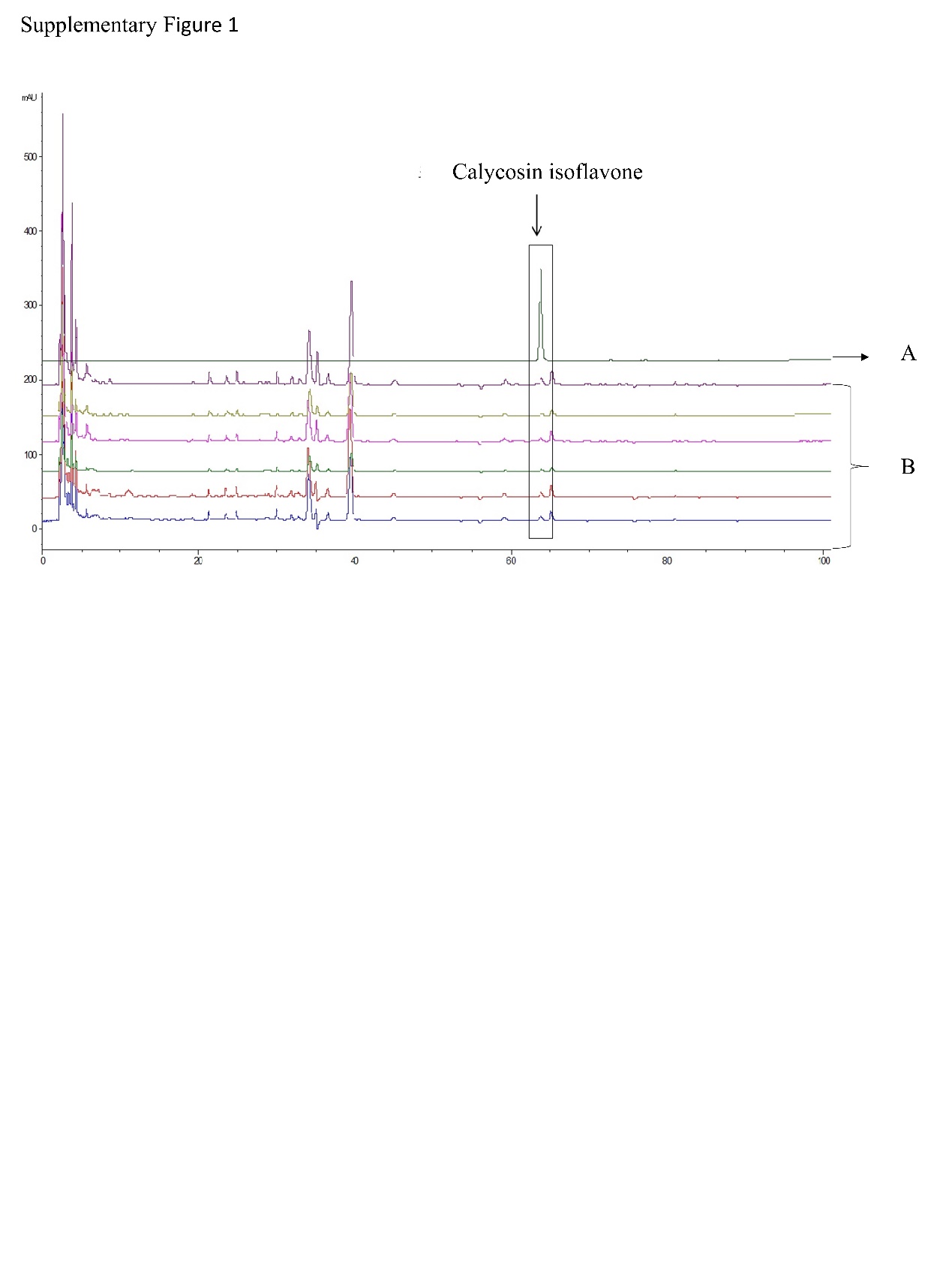


**Supplementary Fig. 1.** A: calycosin isoflavone reference solution; B: YSHS granule solution (batch number: 20201101).
